# Charge inversion under plasma-nanodroplet reaction conditions excludes Fischer esterification for unsaturated fatty acids: a chemical approach for type II isobaric overlap†

**DOI:** 10.1039/d3sc05369e

**Published:** 2023-12-12

**Authors:** Dmytro S. Kulyk, Glib V. Baryshnikov, Purva S. Damale, Simon Maher, Abraham K. Badu-Tawiah

**Affiliations:** a Department of Chemistry and Biochemistry, The Ohio State University 100 West 18th Ave. Columbus OH 43210 USA badu-tawiah.1@osu.edu; b Laboratory of Organic Electronics, Department of Science and Technology, Linköping University SE-60174 Norrköping Sweden; c Department of Electrical Engineering and Electronics, University of Liverpool Liverpool UK

## Abstract

Direct infusion ionization methods provide the highest throughput strategy for mass spectrometry (MS) analysis of low-volume samples. But the trade-off includes matrix effects, which can significantly reduce analytical performance. Herein, we present a novel chemical approach to tackle a special type of matrix effect, namely type II isobaric overlap. We focus on detailed investigation of a nanodroplet-based esterification chemistry for differentiating isotopologue [M + 2] signal due to unsaturated fatty acid (FA) from the monoisotopic signal from a saturated FA. The method developed involves the online fusion of nonthermal plasma with charged nanodroplets, enabling selective esterification of saturated FAs. We discovered that unsaturated FAs undergo spontaneous intramolecular reaction *via* a novel mechanism based on a carbocation intermediate to afford a protonated lactone moiety (resonance stabilized cyclic carbonium ion), whose mass is the same as the original protonated unsaturated FA. Therefore, the monoisotopic signal from any saturated FA can be selectively shifted away from the mass-to-charge position where the isobaric interference occurs to enable effective characterization by MS. The mechanism governing the spontaneous intramolecular reactions for unsaturated FAs was validated with DFT calculations, experimentation with standards, and isotope labeling. This novel insight achieved *via* the ultrafast plasma-nanodroplet reaction environment provides a potentially useful synthetic pathway to achieve catalyst-free lactone preparation. Analytically, we believe the performance of direct infusion MS can be greatly enhanced by combining our approach with prior sample enrichment steps for applications in biomedicine and food safety. Also, combination with portable mass spectrometers can improve the efficiency of field studies since front-end separation is not possible under such conditions.

## Abstract 2

The fusion of nonthermal plasma with charged nanodroplets enables selective esterification of saturated fatty acids, which is utilized to overcome challenges associated with type II isobaric overlap in direct infusion mass spectrometry.

## Graphical abstract

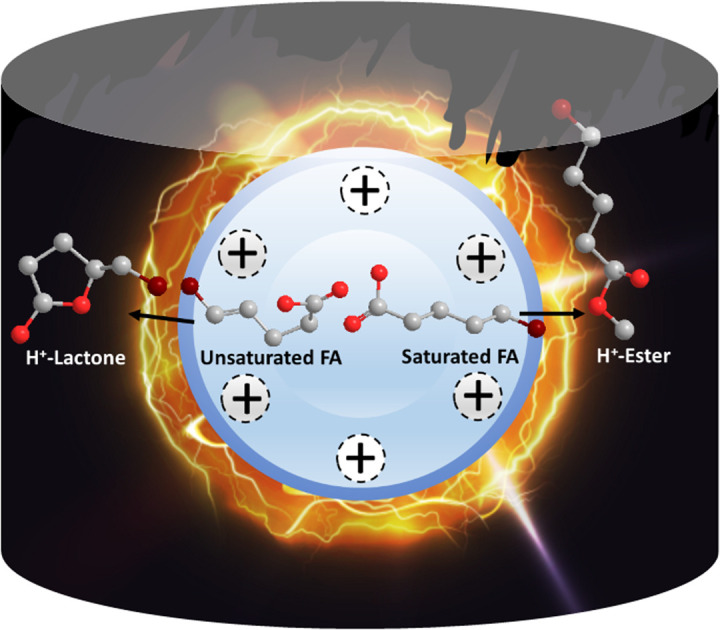

## Introduction

**Scheme 1:**
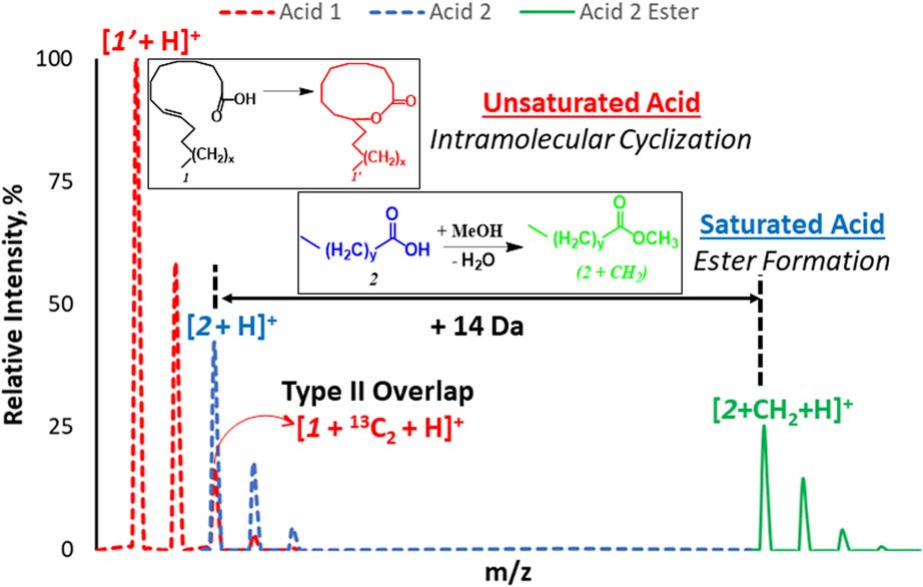
Schematic illustration of type II isobaric overlap phenomenon for acid 1 (unsaturated, red) and acid 2 (saturated, blue) and the use of esterification (green) process to shift mass of saturated acid to a new position that overcomes isobaric overlap.

Fatty acids (FAs) and their esters (including diverse groups of lipids) are important in food safety and disease diagnosis. The detection of FA imbalances (*e.g.*, saturated *versus* unsaturated) can provide insight on health status, especially regarding chronic inflammatory disorders.^1–4^ Shotgun mass spectrometry (MS) is the method of choice for rapid detection of different lipid classes.^5–8^ To be effective, this direct infusion MS method often employs an enrichment step (*e.g.*, *via* solid-phase extraction)^9^ or liquid/liquid extraction^10^ prior to analysis to enable detection of low abundant lipids. While efficient sample preparation methods can help in reducing interference from matrices, such steps are not always available, especially regarding the use of portable mass spectrometers in field application. Also, certain matrix effects may persist such as isotope^11^ and isobaric interferences.^12^ For example, lipid characterization can be complicated by the so-called type II (isobaric) overlap, where the isotopologue [M + 2] peak from unsaturated lipid (*e.g.*, influence of ^13^C on signal of oleic acid, MW 282 Da) interferes with the monoisotopic signal from a saturated lipid (*e.g.*, stearic acid, MW 284 Da, Scheme 1). It has been shown that for low abundant FAs, such signals cannot be differentiated at low resolutions (*R* < 30 000).^13^ The FA derivatives with higher MW will require even higher resolving power to overcome type II isobaric overlap. Such requirement for high resolution has prevented the potential application of portable instruments for analysis of FA mixture and other important metabolites. Therefore, mathematical corrections are often used, especially for quantitative purposes.^14^

We propose a new chemical approach, based on *in situ* nanodroplet-based esterification reaction, that can be applied to shift the main monoisotopic signal from the saturated FA away from the overlapping M + 2 peak due to the presence of unsaturated FA (Scheme 1). The new position of the esterified monoisotopic peak is dictated by the mass of the alcohol used to react with the FA. As part of this long-term goal, the current study seeks to understand why saturated FAs readily undergo esterification in the charged nanodroplet environment while unsaturated FAs appear transparent to the same reaction condition. Such a simple and selective esterification reaction occurring during ionization (nebulizer gas is not required) can have a huge impact on shotgun MS, especially experiments performed on low-resolution instruments and in the field.

FAs exist as anions at neutral or physiological pH and thus ionize well in negative mode electrospray ionization (ESI). Because of this, methods developed for direct infusion MS operate exclusively in the negative-ion mode. But negative ions of FAs do not produce informative fragmentation when subjected to the widely available low-energy collision-induced dissociation (<100 eV) tandem MS (MS/MS) method. While high-energy collisional activation of deprotonated molecular ions [M − H]^−^ produces some product ions for polyunsaturated FAs (albeit weak abundances), minimal dissociation is often observed for saturated FAs. To enable fragmentation along the backbone of FAs, some complex instrumentations are developed, for instance, post-column infusion of a barium ion solution.^15^ FAs can be subjected to reverse derivatization to allow them to be detected in the positive-ion mode, *via* metal attachment^16^ or chemical reactions that labels the FA with a positive charge or a neutral group having high proton affinity.^5,17–19^ Plasma sources have been shown to enable protonation of FAs^20^ in a solvent-free environment but this effects has not been demonstrated for spray-based ionization. Some attempts were made to couple spray-based ionization with nonthermal plasma, but these reports focused on negative-ion mode analysis for epoxidation reaction and subsequent localization of double bonds in unsaturated fatty acids.^21–23^ We discovered that protonated saturated FAs react favorably with alcohols (*e.g.*, methanol and ethanol) to give the corresponding ester under ambient conditions. Here, charged nanodroplets containing the FA were exposed to electrical discharge, enabling an instantaneous reaction with the alcohol, which can be added to the electrospray solvent or introduced into the headspace as a vapor.

## Results and discussion

### Platform for plasma-nanodroplet fusion

**Fig. 1:**
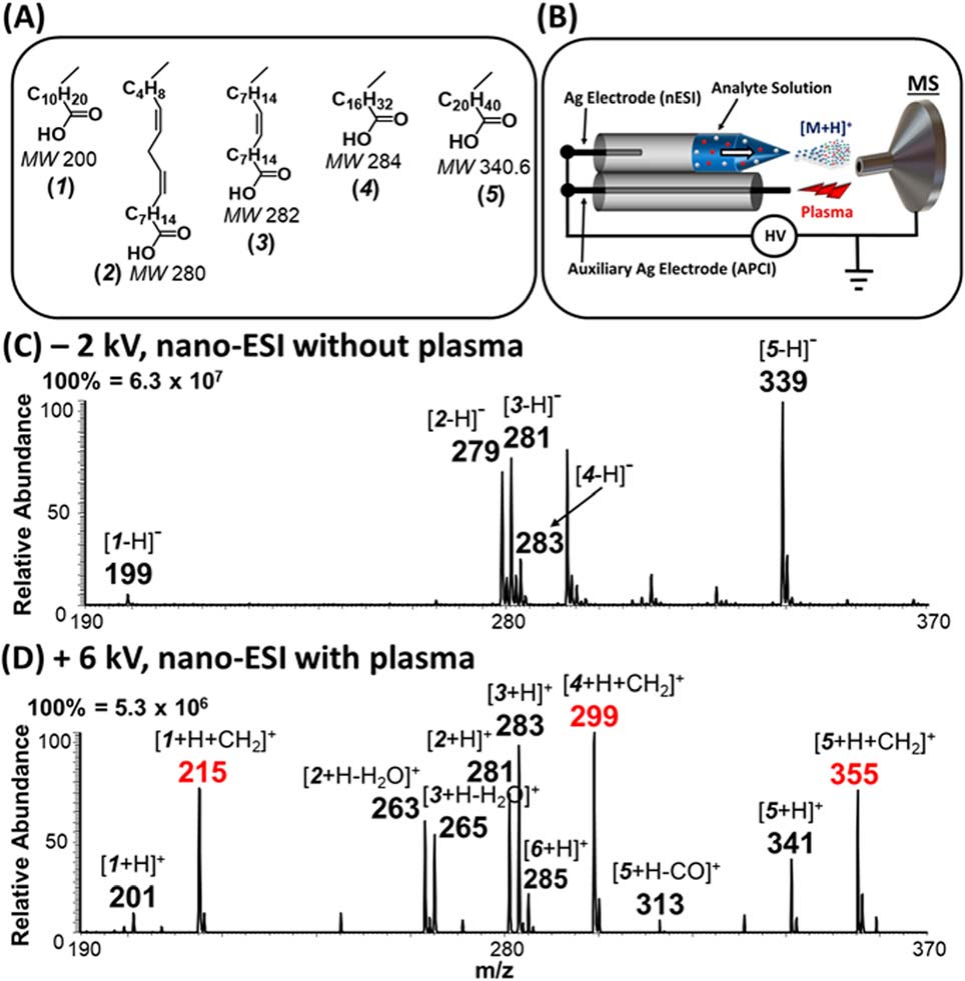
(A) Schematics showing the chemical structures of five fatty acids (FAs): lauric acid (1), linoleic acid (2), oleic acid (3), stearic acid (4), and behenic acid (5). (B) Experimental setup for plasma-nanodroplet fusion and online MS analysis. Nanodroplets are derived from non-contact nano-ESI where the Ag electrode is not in physical contact with analyte solution. Nonthermal plasma is derived from the auxiliary Ag electrode when DC voltage > 4 kV is applied. (C) Typical negative-ion mode mass spectrum recorded following the analysis of a 200 ppm solution containing the five FAs mixture at −2 kV nano-ESI. (D) Typical positive-ion mode mass spectrum of the same FA solution recorded using the plasma-nanodroplet fusion source, using +6 kV applied voltage.

The nanodroplet-based esterification of protonated FAs was achieved using the home-made ion source shown in Fig. 1B. In this case, charged nanodroplets are generated from a non-contact nano-ESI emitter using methanolic solution of FAs. Much higher spray voltages (4–6 kV) can be used in non-contact nano-ESI without damaging the emitter tip.^5^ Corona discharge is generated in the presence of an auxiliary Ag electrode that is also connected to the same direct current (DC) high voltage power supply used to initiate nano-ESI. This allows *in situ* exposure of the solvated gas-phase ions produced by nano-ESI with corona discharge from the auxiliary electrode. This nano-ESI experiment, performed in the presence of electrical discharge (*i.e.*, plasma-nanodroplet reaction system), creates an acidic environment that is necessary for inducing esterification of the FAs in methanol without the addition of acids.

Fig. 1 demonstrates that the fusion of nanodroplets with corona discharge can facilitate the direct analysis of FA mixtures (Fig. 1A). The rapid solvent evaporation from the nanodroplets in the presence of nonthermal plasma (+6 kV) leads to the efficient transfer of analytes into gas phase and formation of highly acidic terminal reagent ions (*e.g.*, H_3_O^+^)^24–27^ in ambient air that act like superacid to enable the protonation of organic acids as shown in Fig. 1D. Note: in the absence of plasma, both nanodroplets and microdroplets failed to produce protonated fatty acids in the positive-ion mode (data not shown). Therefore, instead of analyzing the FAs in traditional negative-ion mode (Fig. 1C), we were able to sensitively detect these acidic analytes in positive-ion mode. Using nanodroplets allow us to perform atmospheric pressure chemical ionization (APCI) analysis without external heating that is typically employed for commercial APCI ion sources. This approach also enables us to use smaller sample volumes.

### Saturated fatty acids undergo instantaneous esterification

**Fig. 2:**
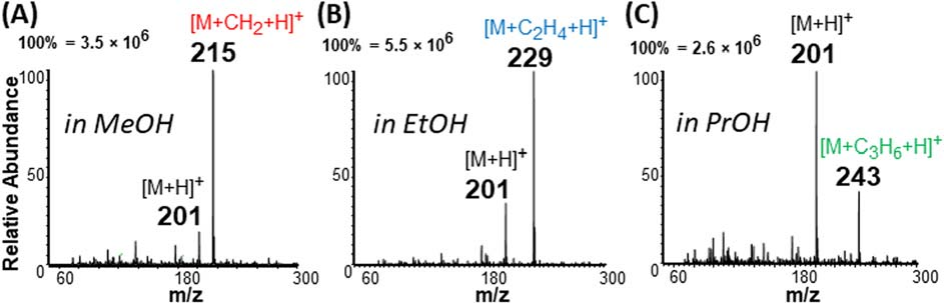
Positive-ion mode spectra for 500 μM lauric acid (M), with solution prepared in (A) methanol (MeOH), (B) ethanol (EtOH), and (C) propanol (PrOH). Esterification occurs for all solvents when plasma-nanodroplet fusion is enabled, yielding expected products at *m*/*z* 215, *m*/*z* 229, and *m*/*z* 243, respectively. Conversion rates for methanol, ethanol, and propanol are 85.7%, 75.8%, and 27.2%, respectively. Conversion rate is defined as the ratio of the absolute ion intensity of a specific ester product relative to the sum of intensities of the fatty acid and the ester derived from this acid.

Interestingly, unsaturated FAs (linoleic (2) and oleic (3) acids), were detected predominantly as protonated species [M + H]^+^, which subsequently lost water to generate [M + H − H_2_O]^+^ ions at *m*/*z* 263 and 265, respectively (Fig. 1D). For saturated FAs (lauric (1), stearic (4), and behenic (5) acids), however, the predominant species were [M + H + 14]^+^, detected at *m*/*z* 215, 299, and 355, respectively, in Fig. 1D. High resolution Orbitrap analysis revealed the elemental composition of the 14 Da to be a methylene group [M + H + CH_2_]^+^ with <3 ppm accuracy. This excluded possible nitrogen insertion or adduction reactions.^28^ Further gas-phase mechanistic investigations were conducted with 23 different carboxylic acids by eliminating the methanol solvent used for nano-ESI (Tables S1, S2 and Fig. S1†). The methanol reagent was brought in as a vapor and could be removed during analysis to evaluate the esterification reaction without interrupting the ionization process. The acids tested include saturated (acetic, propionic, butyric, valeric, hexanoic, decanoic, lauric, myristic, palmitic, stearic, and behenic) and unsaturated (4-pentenoic, 4-hexenoic, *trans*-2-hexenoic, *trans*-3-hexenoic, 5-decenoic, 6-decenoic, *cis*-5-dodecenoic, palmitoleic, linoleic, oleic, vaccenic, and erucic). These experiments revealed that (a) methanol is the source of the methylene (CH_2_) group and (b) the mass of saturated FAs can be shifted to a new mass-to-charge position indicated by the identity of the alcohol used (Fig. 2 and S2†): methanol shifts the peaks by 14 Da while ethanol and propanol shift by 28 Da and 42 Da, respectively. As can be seen in Fig. 2 the yield of the esterification reaction decreased with increasing size of the alcohol. For example, the conversion rate of propyl laurate (*m*/*z* 243, (27.2%)) is 3 times smaller than that for methyl laurate (*m*/*z* 215, (85.7%)). As will be shown later, the esterification reaction can be terminated by using acetonitrile as spray solvent in nano-ESI, which produces simplified spectra in the positive-ion mode.

### Exclusion of methylene insertion

**Fig. 3:**
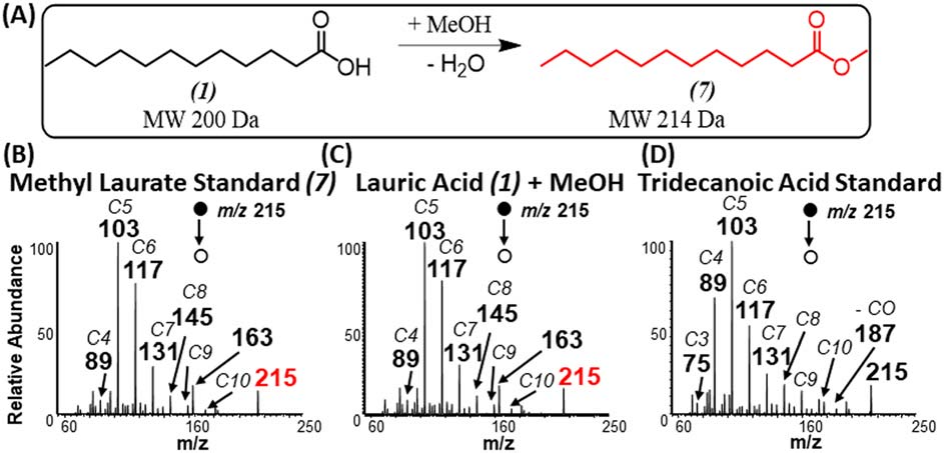
(A) Schematic illustrating esterification reaction between lauric acid and methanol. Positive-ion mode MS/MS analysis of (B) standard methyl laurate, (C) reaction product generated in our experiment from the analysis of lauric acid in the presence of methanol, and (D) standard of tridecanoic acid.

When using methanol as the esterification reagent, a lingering question is related to the fact that methylene attachment to saturated FAs could occur *via* two distinct mechanisms: methylene insertion reaction across a C–C bond,^29^ and esterification reaction. For example, a methylene insertion reaction involving acetic acid will lead to the formation of propionic acid, while the corresponding esterification reaction will afford methyl acetate. Comparison of MS/MS fragmentation patterns from standards of propionic acid and methyl acetate to MS/MS derived from the gas-phase reaction product confirmed the presence of esterification reactions (Fig. S3†). A similar conclusion in favor of esterification was made for propionic, butyric (Fig. S4†) and palmitic (Fig. S5†) acids when each acid reacted with methanol. Further confirmation for esterification was reached when comparing MS/MS from standards of methyl laurate and tridecanoic acid to products formed *in situ* when lauric acid was ionized in the presence of methanol (Fig. 3A–D). That is, the fragmentation pattern for methyl laurate formed during the ionization of lauric acid resembles more closely to MS/MS of standard methyl laurate than MS/MS for tridecanoic acid (consider the abundance of fragments ions *m*/*z* 89, 103, and 117).

**Fig. 4:**
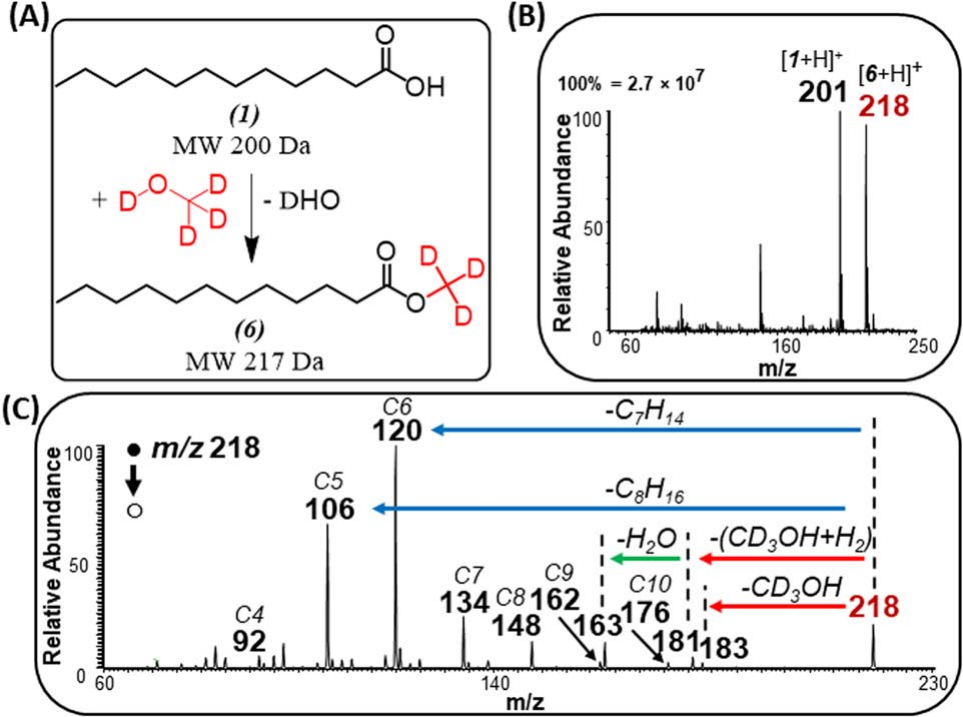
(A) Schematic illustrating gas-phase esterification of lauric acid (1) in the presence of MeOH-D4 vapor. (B) Positive-ion mode full MS analysis showing esterification product (6) at *m*/*z* 218. (C) MS/MS of the esterification product, *m*/*z* 218.

The identity of the ester product was also confirmed by an isotope labeling experiment (Fig. 4) where solid lauric acid was ionized *via* plasma desorption,^25^ followed by *in situ* esterification of the protonated lauric acid with headspace vapor of deuterated methanol (MeOH-D4). We observed an intense peak at *m*/*z* 218, which indicates the formation of deuterated methyl laurate R–CO_2_–CD_3_ (MW 217 Da). Note: unlike saturated FAs, unsaturated FAs were not reactive toward MeOH-D4, indicating some structural differences between the two fatty acids (discussed below).

### Factors influencing reactivity of saturated *versus* unsaturated fatty acids

Having established esterification reactions under the plasma-nanodroplet spray conditions, the next important question to answer was why esterification can occur more favorably for saturated FAs but not for unsaturated FAs. First, we note that no external acid source was used. This effect is unexpected because Fischer esterification of FAs with alcohol is a slow endothermic process when conducted in bulk solution-phase. This reaction requires heating in the presence of concentrated acids (*e.g.*, H_2_SO_4_) with a high activation energy. Upon dissolution in the alcohol, strong acids produce the conjugate acid of the alcohol that serves as acid catalyzer. The ability of the plasma-nanodroplet fusion system to facilitate esterification in the absence of external acid supports the suggestion that the reagent ions involved in positive-ion mode are highly acidic (*e.g.*, proton affinity (PA) of H^+^(H_2_O) is 725.6 kJ mol^−1^).^30^

#### Product stability and the importance of C=C bond position

**Scheme 2:**
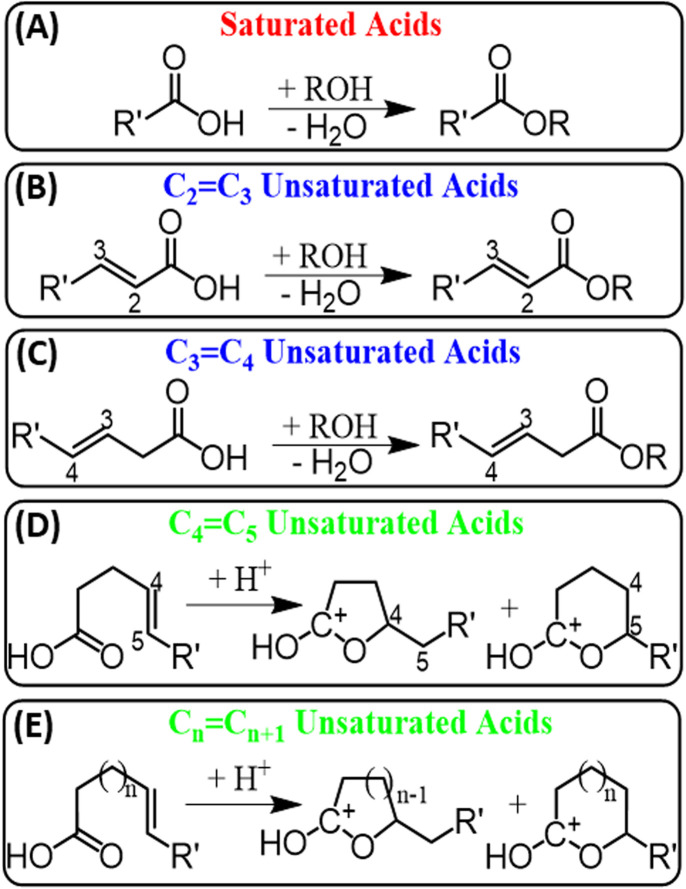
Schematic summarizing fatty acids behaviour in the presence of alcohols under the plasma-nanodroplet fusion conditions where Fischer esterification is observed for (A) saturated acids, (B) unsaturated acids with C=C bond at position 2, and (C) at position 3. Unsaturated acids with C=C bonds (D) at position 5 and (E) farther than C_5_ show intramolecular cyclization leading to the formation of carbonium ions.

Several factors have been considered to understand why esterification occurs more favorably for saturated FAs, including stability of esterification product, difference in proton affinities, steric hindrances, kinetics, and concurrent plasma reactions. For example, although some report show methyl esters of unsaturated FAs to be thermally unstable,^31^ all attempts to detect the desired esterification product for unsaturated FAs in our experiments failed, including the use of ambient temperature, minimal breakdown voltage (4 kV), and minimal number of double bonds (one). Then, we decided to change the position of the C=C bond in the unsaturated FAs, by moving the C=C bond closer to the carboxylic head group. No esterification was observed until the C=C bond was placed three (3-hexenoic acid) or two (2-hexenoic) carbons away from the carboxylic group (Scheme 2B, C and Table S2†). The stability of methyl esters can be expected to decrease when C=C bond is located near the ester group due to their increased reactivity. However, we observed the opposite effect where the ester product was not detected for FAs with C=C bond far away from the carboxylic head group. This observation led us to conclude that the stability of methyl esters of unsaturated FAs is not the key factor that controls their reactivity with alcohols.

#### Proton affinity and reaction kinetics are not major factors

Since protonation is the crucial step in Fischer esterification, our next investigation was related to probable difference in proton affinities for unsaturated *versus* saturated FAs. However, our analysis revealed that any differences in proton affinity cannot lead to selective suppression of unsaturated FAs toward esterification (see ESI† for details). Another possible explanation for the difference in reactivity between saturated and unsaturated FAs can be related to kinetics. For instance, esterification of unsaturated FAs may be too slow to be detected by the mass spectrometer during the microsecond timescale of the plasma-nanodroplet fusion experiment. However, this timescale argument does not hold up either because esterification is observed for unsaturated FAs having C=C bonds very close to the carboxylic head group. Why, then, is esterification hindered for FAs when the C=C bond is located farther from the carboxylic head group?

#### Dehydration is not an important competitive reaction

The logical last option left to explain the reactivity difference was to assume a concurrent/competing reaction that is either thermodynamically or kinetically more favorable than esterification. Since mass spectra of unsaturated FAs recorded in the presence of alcohol do not show any extra peaks related to condensation reactions, any potential concurrent reaction products can only be a structural isomer of the acid and/or its dehydration product as detected in our experiment. The dehydration pathway was observed for all 12 unsaturated acids tested (Table S2†), but their relative abundance was always less (often significantly less) than the abundance of the protonated fatty acid. This indicates that the reaction leading to water loss cannot substantially outcompete esterification of unsaturated fatty acid if the latter reaction was possible. Therefore, we proposed that another competing reaction must exist to prevent esterification of unsaturated FAs, except that this reaction could produce a structural isomer of the FAs whose mass cannot be differentiated from the original acid *via* MS. Unfortunately, MS/MS data obtained before and after applying alcohol to FA did not show distinct fragmentation patterns, which rules out the potential for significant rearrangement in the resultant isomeric product.

### Intramolecular reactions for unsaturated fatty acids

Considering fatty acid chemistry, a good reaction candidate may include intramolecular cyclization to give the corresponding lactone of the starting fatty acid, with no change in molecular weight. Since this reaction is also an esterification process, it can be spontaneously favored under the fast plasma-nanodroplet reaction conditions to prevent intermolecular esterification with an external alcohol. Since intramolecular esterification reactions leading to lactones traditionally involve a hydroxycarboxylic acid intermediate,^32^ we anticipated oxidization of the double bond can make this mechanism possible. However, all attempts to detect the hydroxycarboxylic acid intermediate failed (Fig. S6†). In addition, we conducted DFT calculations, which showed that the last (deprotonation) step in the mechanism involving hydroxycarboxylic acid intermediate is thermodynamically disfavored with +50.4 kcal mol^−1^ activation energy (Mechanism II, Fig. S7†).

### Protonated lactone formation *via* carbocation intermediate proved by DFT calculation

**Fig. 5:**
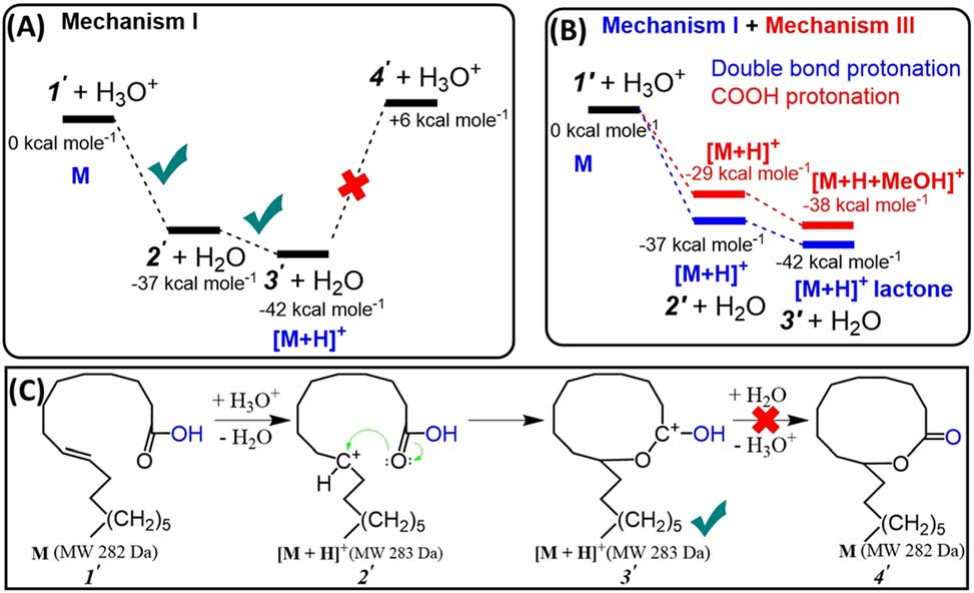
(A) Free energy diagram of gas-phase intramolecular cyclization of oleic acid. (B) Comparison of DFT calculations for the energetics involved in the protonation of the double bond in oleic acid *versus* the protonation of the carboxyl group. (C) Scheme showing stepwise mechanism for intramolecular cyclization of unsaturated fatty acids as discovered in this study (Mechanism I). See alternative Mechanism II (lactone formation through hydroxycarboxylic acid) in ESI.†

All these insights motivated us to consider a novel, energetically more favorable intramolecular mechanism for probable lactone formation. This mechanism involves the protonation of the C=C bond present in the unsaturated FA, which subsequently reacts with the carboxylic head group. Note: both double bond carbons for oleic acid can be equally protonated (C_9_ or C_10_). Our DFT calculations (Fig. S8†) showed that PAs of these carbons are similar (PA (C_9_) = 810.1 kJ mol^−1^, PA (C_10_) = 809.2 kJ mol^−1^). This should allow the formation of 10- and 11-membered ring lactones with equal probability. Fig. 5A and C reflect the proposed three step mechanism for oleic acid, namely: (a) C=C bond protonation at C_9_ to form carbocation (−37 kcal mol^−1^), (b) cyclization *via* nucleophilic attack of carbocation by carbonyl oxygen (−5 kcal mol^−1^) to form 10-membered ring carbonium ion, and (c) deprotonation (+48 kcal mol^−1^). As can be seen (Fig. 5A), the last (deprotonation) step is significantly endothermic. This suggests that the intramolecular reaction terminates at the penultimate step (3′; Fig. 5C), yielding the resonance stabilized carbonium ion (protonated lactone) that is readily detected by the mass spectrometer. Fig. 5B compares free energy diagrams of the first two steps involved in intramolecular cyclization for the mechanism proposed in this work (Mechanism I) and the traditional Fischer esterification mechanism (Mechanism III), obtained by DFT calculations (see more details in ESI, Fig. S9†). The results clearly prove that double bond protonation and consequently intramolecular cyclization is more energetically favorable (by 8 kcal mol^−1^) than protonation of carbonylic group.

The typical mechanism proposed for acid-catalyzed synthesis of lactones from unsaturated fatty acid involve the migration of C=C bond to a position that favors stable ring formation.^33^ Since we observed a strong dependence of C=C bond position on reactions observed, we do not expect the C=C bond migration step to be important in our plasma-nanodroplet reaction condition. This might be due to the rapid timescale of our experiment, which will not support the slow endothermic process of C=C bond migration. Also, our DFT calculations confirmed that the stabilities of 6-membered 
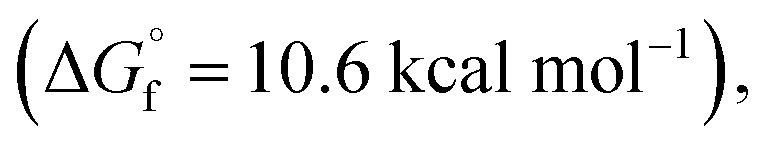
 10-membered 
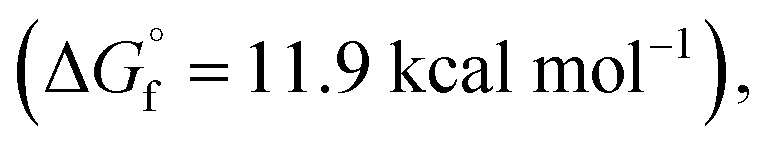
 and 11-membered 
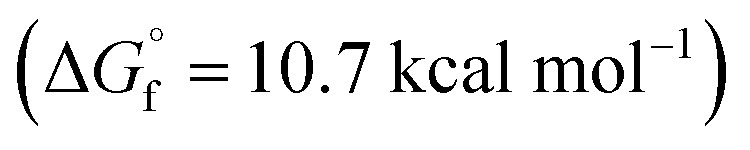
 lactones formed from oleic acid in gas phase are comparable. This data does not support thermodynamical necessity to move the C=C bond to any specific position to favor intramolecular reaction under the plasma-nanodroplet reaction conditions.

### Experimental evidence: unsaturated FAs behave as carbonium ions

**Fig. 6:**
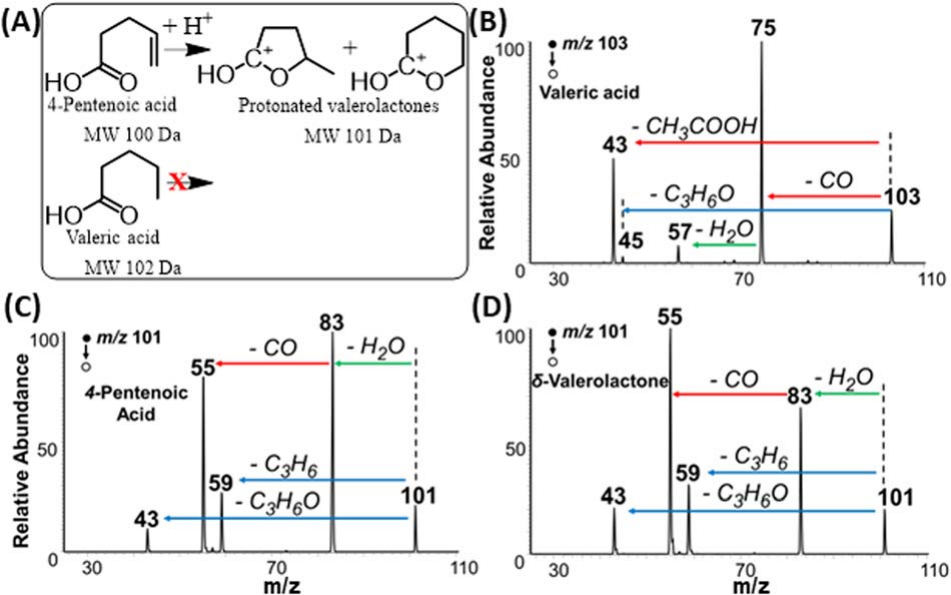
(A) Schematics displaying the intramolecular cyclization of 4-pentenoic acid to give cyclic carbonium ions while valeric acid cannot be cyclized. Positive-ion mode MS/MS spectra of standards of (B) valeric acid, (C) 4-pentenoic acid, and (D) δ-valerolactone. 4-Pentenoic acid converts into 5- and 6-membered cycled carbonium ions, showing identical MS/MS spectra (C and D).

**Fig. 7:**
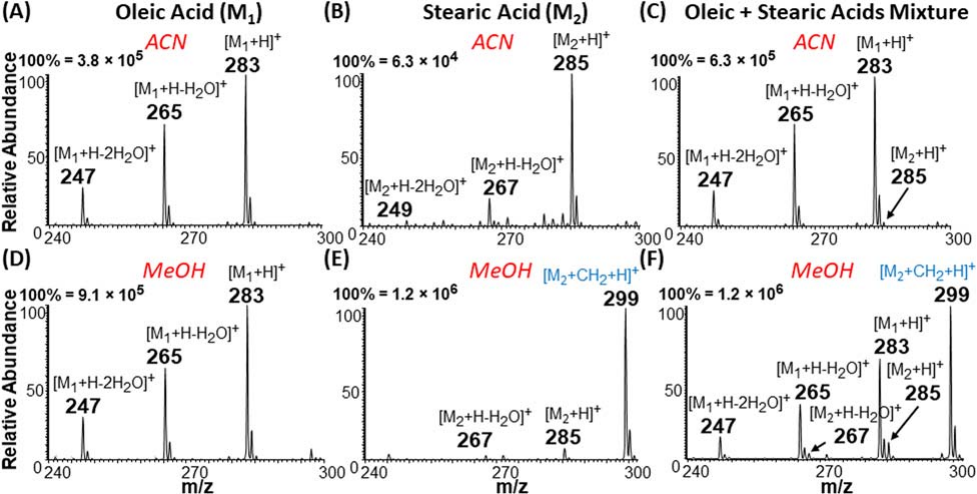
Positive-ion mode full MS analysis of 500 μM each of (A and D) oleic acid in (A) acetonitrile (ACN) and (D) methanol; (B and E) stearic acid in (B) acetonitrile and (E) methanol; and (C and F) equimolar mixtures (500/500 μM) of oleic and stearic acids in (C) acetonitrile and (F) methanol. Each analyzed solution was tested with the plasma-nanodroplet fusion platform using +6 kV spray voltage.

The proposed Mechanism I (Fig. 5C) was also proved experimentally. Tandem MS data in Fig. 6 (also Fig. S10†) demonstrates that fragmentation patterns for standard 4-pentenoic acid (unsaturated acid, Fig. 6C) and standard δ-valerolactone (lactone, Fig. 6D) are identical when analyzed under the plasma-nanodroplet reaction condition. Both compounds show significant water loss followed by the elimination of CO in a sequential fashion. In contrast, MS/MS of protonated valeric acid (saturated acid, Fig. 6B) differs significantly in which a competitive fragmentation is observed between CO loss and acetic acid elimination. In other words, protonated valeric acid maintains its acidic nature in the gas-phase while 4-pentenoic acid (unsaturated acid) undergoes intramolecular esterification to change its acidic nature into a corresponding carbonium ion, although molecular weight remains the same (Fig. 6A). Thus, MS/MS analysis of protonated 4-pentenoic acid is indeed an analysis of protonated valerolactone (either as major product γ-valerolactone or minor product δ-valerolactone) since pentenoic acid will undergo spontaneous intramolecular reaction. Identical MS/MS spectra were also obtained for dehydrated 5-hydroxydecanoic acid and δ-decalactone (Fig. S6†) that proves spontaneous intramolecular esterification of hydroxycarboxylic acids under our plasma-nanodroplet analysis conditions. In general, all lactones and majority of unsaturated FAs (Table S1†) behave similarly in full MS and MS/MS in that they lose two water molecules while saturated FAs behave differently to show fragments originating from the carboxylic group and/or the fatty acyl chain (Fig. S11†). For saturated FAs, spontaneous water loss is not a major event, as shown in the full MS analysis (Fig. 1D, 7, S5, S11, Tables S1 and S2†).

Of all 12 unsaturated FAs tested, only two of them fragmented similarly to saturated FAs. These are 3-hexenoic acid and 2-hexenoic acid, which are the same acids that underwent intermolecular esterification with alcohols (see Scheme 2B and C). This behavior is ascribed to two possible reasons: firstly, lactones with 3- or 4-membered rings (α-lactones and β-lactones) are unstable, which can open the lactone ring to give the corresponding acid. Secondly, when we performed DFT calculations to compare PAs of carbonyl, hydroxyl, and C=C bond of FAs, we discovered that the C=C bond always showed higher proton affinity, unless it is located after the second or third carbons (Fig. S8†). Therefore, there is a high likelihood that unsaturated fatty acids with C=C bond located at C_2_ and C_3_ do not undergo intramolecular cyclization at all, and thus maintain their acidic nature during analysis by the plasma-nanodroplet fusion platform. Note: no catalyst or heat was used for our plasma-nanodroplet derivatization procedure, which is markedly different from conventional bulk-phase methods.

### Unsaturated FAs ionize better because they are changed into cyclic carbonium ions

The apparent difference in chemical nature of saturated FA (behaves as acid) and unsaturated FA (behaves as cyclic carbonium ions) under the plasma-nanodroplet analysis condition is responsible for inducing markedly different ionization efficiencies for the two species. For example, the probability of ionizing saturated fatty acids in positive-ion mode is at least one order of magnitude smaller than ionizing unsaturated fatty acids in the positive-ion mode as protonated species regardless of solvent used (Fig. 7). This is not surprising because of the resonance stabilized cyclic carbonium ions derived from unsaturated FA. The presence of positive charge inherently provides higher ionization efficiency compared with the need to protonate the carboxylic acid functional group in saturated FAs. Besides, cyclic esters have higher proton affinity than acids (Fig. S8†). Therefore, the esterification reaction achievable under the plasma-nanodroplet condition can reduce this ionization inefficiency by converting the acid to an ester moiety. This effect is illustrated for equimolar analysis of oleic acid and stearic acid in different solvents. Esterification occurs in methanolic solvent, which provides opportunities for the detection of saturated stearic acid (*m*/*z* 299), in the presence of oleic acid (Fig. 7F). No esterification occurs in acetonitrile solvent, and thus stearic acid is significantly suppressed in the presence of oleic acid (Fig. 7C).

### Solving the type II isobaric challenge

**Fig. 8:**
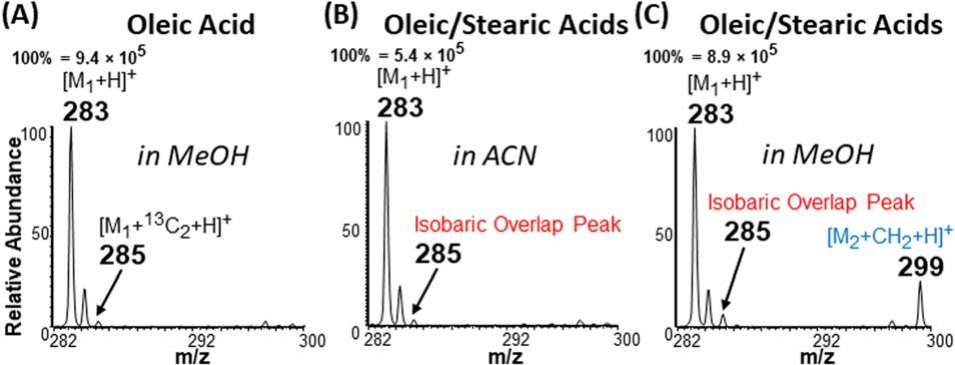
Demonstration of the type II isobaric overlap phenomenon and how the effect is overcome by esterification. Positive-ion mode mass spectra obtained from the analysis of (A) 500 μM oleic acid (M_1_) in methanol, (B) 500 μM oleic acid with 10% stearic acid (M_2_; 50 μM) prepared in acetonitrile (no esterification), and (C) 500 μM oleic acid with 10% stearic acid (50 μM) prepared in methanol (esterification occurs). Isobaric overlap peak is a mixture of [M_1_ + ^13^C_2_ + H]^+^ and [M_2_ + H]^+^ species occurring at *m*/*z* 285. During esterification the signal due to stearic acid is shifted to *m*/*z* 299 enabling its characterization.

With these insights, we applied the plasma-nanodroplet fusion experiment to tackle challenges associated with FA detection in the presence of type II isobaric overlap (Scheme 1). It is clear why it could be extremely difficult to detect a low-abundant saturated FA signal in the presence of unsaturated FA (due to isobaric overlap and ionization efficiency). However, the presence of the saturated FA can now be confirmed by selectively shifting its mass away from the M + 2 position *via* esterification. We demonstrated this capability by analyzing oleic acid containing 10% stearic acid (Fig. 8). The results indicate that MS (Fig. 8A, B and S12†) and MS/MS (Fig. S13†) analyses failed to differentiate between the two isobaric species at *m*/*z* 285: [M + ^13^C_2_ + H]^+^ from oleic acid and [M + H]^+^ from stearic acid. By applying the plasma-nanodroplet platform, selective esterification was induced to shift the stearic acid signal by 14 Da to give the new peak at *m*/*z* 299 (Fig. 8C). The signal due to esterification was not detected when the FAs were prepared in acetonitrile instead of methanol, in which case the presence of stearic acid could not be confirmed due to significant isobaric overlap at *m*/*z* 285 (Fig. 8B). Calibration curve for online esterification of stearic acid in the presence of oleic acid was constructed (Fig. S14†) where the methyl stearate ion (*m*/*z* 299) intensity at different concentrations of stearic acid analyte (5–500 μmol L^−1^) was compared to that of oleic acid (500 μmol L^−1^, *m*/*z* 283). The FAs solutions were prepared in methanolic solvent. Excellent linearity (*R*^2^ = 0.999) and repeatability (RSD ≤ 10%) were achieved.

### Charge inverted FAs show abundant fragmentation

**Fig. 9:**
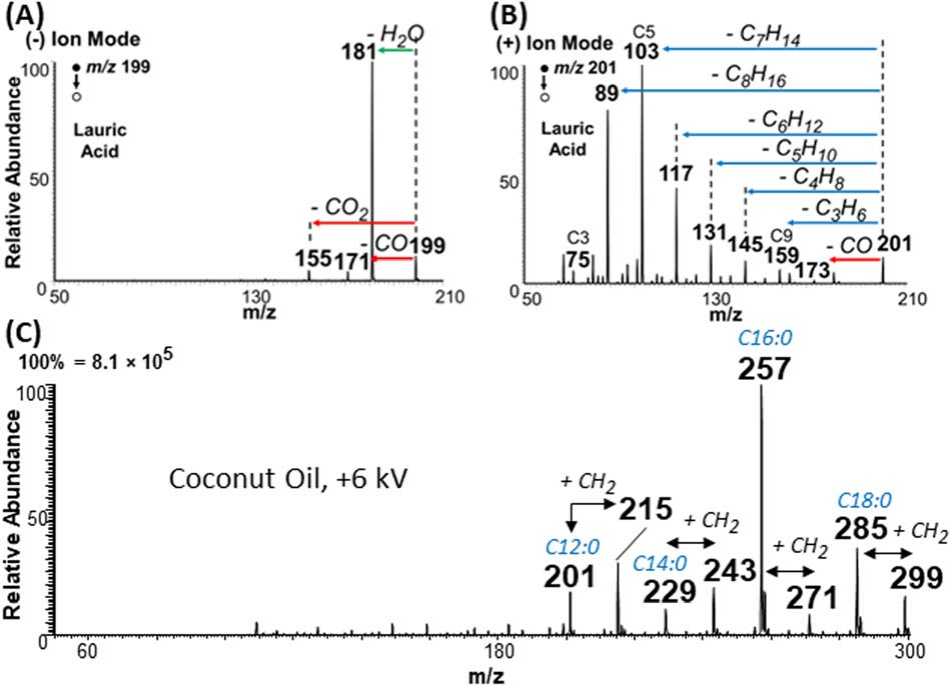
Comparison of tandem MS data (CID) in (A) negative- and (B) positive-ion modes for lauric acid at *m*/*z* 199 and 201, respectively. (C) Positive-ion mode mass spectrum showing the analysis of complex coconut oil diluted in MeOH using the plasma-nanodroplet fusion setup with +6 kV DC voltage applied.

Aside from overcoming type II overlap, another important feature of the plasma-nanodroplet fusion experiment is related to the abundant fragmentation available for FAs when they are subjected to charge inversion and analyzed in positive-ion mode. Example is illustrated for lauric acid in Fig. 9A and B. As can been seen, MS/MS for negative-ion mode using deprotonated ions reveals information only about the polar carboxyl group, while MS/MS of protonated ions in the positive-ion mode provides rich information about the fatty acyl chain. This observation was reproduced for many other FAs (Fig. S15–S19†). The fragmentation pattern of esters in positive-ion mode is remarkably similar to that of protonated FAs in that both species displayed sequential loss of ethylene. The only difference, however, is related to water loss for FAs *versus* MeOH loss for esters (Fig. S20 and S21†). This suggests that the MS/MS of the ester can be used to characterize saturated FAs whose signals are overwhelmed by type II isobaric overlap.

### Application to complex mixture analysis

We applied the plasma-nanodroplet platform to analyze coconut oil from *Cocos nucifera*. Our main objective was to evaluate the feasibility of achieving esterification in complex mixtures. We choose coconut oil because the nutritional value of this oil has been a source of considerable debate.^34^ Due to its importance, the chemical composition of coconut oil has been characterized. However, the high fraudulent activities (*e.g.*, adulteration) related to this oil makes it necessary to develop simple methods that can monitor quality. Without prior separation, our charge inversion method enabled several important data to be collected for oil authentication without modifying the existing mass spectrometer.

For example, when applying the conventional negative-ion mode analysis at low DC voltage (−2 kV, in the absence of plasma), we detected peaks that appear to corroborate the presence of the expected saturated FAs (Fig. S22A:† caprylic, capric, lauric, myristic, palmitic, and stearic acids). Signal corresponding to two unsaturated FAs (oleic and linoleic acids) were also detected. As expected, MS/MS of the negative ions did not provide sufficient information to fully confirm the structure of the species detected (data not shown). Conventional positive-ion mode analysis at low voltage (+2 kV, no plasma induced) showed (Fig. S23A†) the presence of larger molecular lipids (sodium adducts, also known),^35^ but not the corresponding FA composition (since charge inversion is not possible at low voltage). Upon the induction of nonthermal plasma, however (by applying +6 kV DC voltage), we detected 6 FAs in positive-ion mode, including the corresponding esters of the four major saturated FAs found in coconut oil (Fig. 9C). With these charged inverted species, we were able to fully characterize FA composition using the conventional collision induced dissociation MS/MS method (Fig. S24†). Negative plasma (*i.e.*, plasma-nanodroplet reaction at −4 kV) did not significantly change the profile of FAs or lipids detected (Fig. S25†).

To confirm that the large molecular weight species detected in low spray voltage (+2 kV, Fig. S23A†) were related to FA composition of the coconut oil, we performed basic hydrolysis (see details for hydrolysis procedure in ESI†). These larger lipids were not detected when the hydrolyzed coconut oil was analyzed at +2 kV (Fig. S23B†). Interestingly, the relative intensity of some FAs detected at +6 kV increased in the positive-ion mode, which allowed the corresponding esters of all six saturated FAs to be detected (Fig. S26†), including the least abundant caprylic acid (C8:0, whose concentration in coconut oil has been reported to be 0.7%,^36^ corresponding to an estimated concentration of 6 ppm in the sample solution). Similarly, the abundance of FAs detected in the hydrolyzed coconut oil increased (10×) when analyzed in negative-ion mode at low (−2 kV) voltage (Fig. S22B†). The FAs and lipid profiles detected here in coconut oil are in good agreement with what has been reported in recent literature.^35,36^

## Conclusions

In conclusion, a new chemical approach has been established that has potential to reduce the effects of type II isobaric overlap for a more efficient shotgun mass spectrometry analysis of fatty acids. This technology is based on the fusion of nonthermal plasma from corona discharge with nanodroplets from nano-electrospray, which was found to facilitate the selective esterification of saturated fatty acids without the need for a catalyst or heat. The mechanism governing this selective esterification was fully investigated. We observed that unsaturated fatty acids prefer to proceed under spontaneous intramolecular reactions to form the corresponding cyclic carbonium ion through a carbocation intermediate. This reaction did not require the migration of the C=C bond. This mechanistic insight was validated with DFT calculations, experimentation with standards, and isotope labeling. Based on the alcohol used (methanol, ethanol, or propanol), the signal from the saturated fatty acid was shifted to a new mass-to-charge position corresponding to the esterified product, which eliminated the overlap from the carbon-13 isotopologue [M + 2] signal. The reproducibility of the approach was tested by analyzing more than 20 fatty acids. Among all unsaturated fatty acids, those with C=C bonds located at C_2_ and C_3_, are the only species found to undergo Fischer esterification. Although such unsaturated fatty acids are not dominant in nature, they can be differentiated from saturated fatty acids by mass difference of corresponding alcohol esters in MS spectrum.

The plasma-nanodroplet fusion experiment also allowed fatty acids (saturated and unsaturated) to be detected in both negative- and positive-ion modes. The intramolecular cyclization observed for unsaturated fatty acids efficiently boosts their ionization efficiency in the positive-ion mode. Tandem MS of the charge inverted protonated [M + H]^+^ species (positive-ion mode) were found to give abundant fragment ions along the acyl carbon chain. This information is missing from MS/MS derived from the conventional methods utilizing deprotonated [M − H]^−^ ions. Interestingly, tandem MS of the methyl ester derived from saturated fatty acids are remarkably similar to that of the protonated fatty acids. This enabled low abundant species, which would otherwise experience type II isobaric overlap at [M + H]^+^ signal level to be effectively characterized after esterification using the [M + CH_2_ + H]^+^ signal. This feature was successfully demonstrated *via* the characterization of 10% stearic acid in oleic acid solution. We further tested the robustness of the approach by analyzing complex coconut oil in which we detected the charge inverted species of all expected fatty acids, including their corresponding esterified products. Collectively, our results showcase how a simple but effective chemical approach could be employed to improve the performance of shotgun mass spectrometry. There is high potential for this method to also improve the performance of portable mass spectrometers, especially when combined with prior enrichment steps.

## Data availability

All data are available from the corresponding author upon request.

## Author contributions

A. K. B.-T. and D. S. K. conceptualized the idea and planned the experiments. D. S. K. performed all experiments and data analysis with assistance of P. S. D. DFT calculations were carried out by G. V. B. with assistance of D. S. K. The first draft of manuscript was written by A. K. B.-T. and D. S. K., which was improved *via* discussions/contributions from all authors.

## Conflicts of interest

There are no conflicts to declare.

## Supplementary Material

SC-015-D3SC05369E-s001
