# Supplementary material for: Charge inversion under plasma-nanodroplet reaction conditions excludes Fischer esterification for unsaturated fatty acids: a chemical approach for type II isobaric overlap
Source: Chem Sci. 2023 Dec 12;15(3):914–22. doi: 10.1039/d3sc05369e (PMC10793210; doi:10.1039/d3sc05369e)
Supplement: SC-015-D3SC05369E-s001 [file SC-015-D3SC05369E-s001.pdf]

Electronic Supplementary Material (ESI) for Chemical Science.
This journal is © The Royal Society of Chemistry 2023

SUPPORTING INFORMATION

Charge Inversion Under Plasma-Nanodroplet Reaction Conditions Excludes Fisher Esterification for Unsaturated Fatty Acids: A Chemical Approach for Type II Isobaric Overlap

*Dmytro S. Kulyk[1], Glib V. Baryshnikov[2], Purva S. Damale[1], Simon Maher[3], and Abraham K. Badu-Tawiah[1]**

[1] Department of Chemistry and Biochemistry, The Ohio State University, Columbus OH, 43210 (USA)

[2] Department of Science and Technology, ITN, Linköping University, Norrköping 601 74 (Sweden)

[3] Department of Electrical Engineering and Electronics, University of Liverpool, Liverpool (UK)

*Corresponding Author:

Prof. Abraham Badu-Tawiah: Tel.: Fax: (614) 929-4276, and Fax: (614) 929-1685

Email: badu-tawiah.1@osu.edu

Supporting information is summarized in the table below

Topic	Title of Topic	Page
**Topic 1**	Experimental section	S3
**Topic 2** (Tables S1, S2, Figure S1)	Full MS analysis of saturated and unsaturated fatty acids	S4
**Topic 3** (Figure S2)	Esterification of saturated fatty acids by ethanol	S6
**Topic 4** (Figures S3-S5)	Determination of the structure of $[M+H+14]^+$ product	S7
**Topic 5** (Figures S6, S7)	Lactone formation from hydroxycarboxylic acids	S10
**Topic 6** (Figure S8)	Proton affinity discussion and calculations	S12
**Topic 7** (Figure S9)	Fisher esterification mechanism	S13
**Topic 8** (Figure S10)	Intramolecular cyclization of unsaturated fatty acids	S14
**Topic 9** (Figure S11)	Plasma fragmentation of saturated and unsaturated fatty acids	S15
**Topic 10** (Figure S12, S13)	Isobaric overlap experiments	S16
**Topic 11** (Figure S14)	Calibration curve for online esterification	S17
**Topic 12** (Figure S15-S21)	CID fragmentation of fatty acids and their esters	S18
**Topic 13** (Figure S22-S26)	Coconut oil analysis	S21
**Topic 14**	References	S24

1. Experimental section

Mass spectrometry. Thermo Fisher Scientific Velos Pro LTQ and LTQ Orbitrap (for high-resolution detection) mass spectrometers (San Jose, CA, USA) were used for analysis and data collection. Unless otherwise stated, MS parameters applied were as follows: 400 °C capillary temperature, 5 mm distance from an ion source to MS inlet, 3 microscans, 100 ms ion injection time, 60% S-lens voltage for Velos Pro LTQ and 55V Tube Lens voltage for LTQ Orbitrap. Spray voltage in the range of 1.5 − 2 kV was used for contact and noncontact nano-ESI MS analysis. To induce plasma during nano-ESI, 6 kV was used, which enabled plasma-nanodroplet fusion. Headspace vapor analysis (in the absence of nano-ESI) was also performed using 6 kV applied to a silver (Ag) electrode in close proximity to the sample to be analyzed (See Fig S1A). Solids were also analyzed in a similar manner, by applying 6 kV to the Ag electrode, in the absence of nano-ESI. Mass spectra were recorded for at least 30 s, yielding an average of 300 individual scans. Data collection and processing for MS were performed with Thermo Fisher Scientific Xcalibur 2.2 SP1 software. Unless otherwise mentioned, Tandem MS with collision-induced dissociation (CID) was used for structural characterization of analyte. We used 30% (manufacturer's unit) and 1.5 Th (mass / charge units) of normalized collision energy and isolation window for CID experiments, respectively.

Materials and Reagents. Acetic acid (≥99.7%), behenic (docosanoic) acid (99%), chloroform (≥99%), coconut oil from *Cocos nucifera*, *δ*-decalactone (≥98.0%), 3,4-dihydro-2*H*-pyran (97%), dodecanoic (lauric) acid (≥99.5%), *cis*-5-dodecenoic acid (99%), *trans*-2-hexenoic acid (99%), *trans*-3-hexenoic acid (97%), 5-hydroxydecanoic acid sodium salt (≥97%), linoleic acid (≥99%), *α*-linolenic acid (≥99%), methanol (≥99.9%), methyl *trans*-3-hexenoate (≥97%), myristic acid (99.5%), oleic acid (≥99%), palmitic acid (≥99%), 4-pentenoic acid (97%), stearic acid (95%), tridecanoic acid (≥98%), *cis*-vaccenic acid (≥97%), and *δ*-valerolactone were all purchased from Sigma-Aldrich (St. Louis, MO, USA). Butyric acid (≥99%), decanoic acid (≥98.0%), formic acid (88%), hexanoic acid (≥98%), methyl acetate, potassium hydroxide (≥85%), propionic acid (≥99%), and valeric acid (≥99%) were supplied by Fisher Scientific (Pittsburgh, PA, USA). Palmitoleic acid (99%) was acquired from Acros Organics (Geel, Belgium). Erucic acid was provided by Agilent Technologies (Santa Clara, CA, USA). 4-Hexenoic acid (*cis* + *trans*) was obtained from Santa Cruz Biotechnology (Dallas, TX, USA). 5-(6)-Decenoic acids mixture was supplied by ArrakisTek (Fairfield, NJ, USA). Methyl laurate (99%) was bought from Frontier Scientific (Logan, Utah, USA). Ethanol 200 Proof (99.5%) was purchased from Decon Laboratories (King of Prussia, PA, USA). Borosilicate capillaries (ID 1.17 mm) were supplied by Sutter Industries (Novato, CA, USA).

2. Full MS analysis of saturated and unsaturated fatty acids.

Table S1. Full MS analysis and esterification of saturated fatty acids achieved using methanol vapor and the vapors of the carboxylic acids in the presence of positive and negative corona discharge. In the Table (-) and (+) denote fragments detected in negative and positive-ion mode. Fragments in (-) and (+) column are shown in order of decreasing MS relative intensity. Mass accuracy (in ppm) data are provided for high resolution MS data for some ester products.

#	Acid	Formula	Carbon #	MW	Esterification	Fragments (-)	Fragments (+)
1	Formic	H-COOH	1	46	No	-	CH_2
2	Acetic	CH_3-COOH	2	60	Yes	-	H_2O, CO
3	Propionic	CH_3-CH_2-COOH	3	74	Yes	-	H_2O, HCOOH
4	Butyric	CH_3-$(CH_2)_2$-COOH	4	88	Yes	C_2H_6, HCOOH	HCOOH, $2H_2O$, H_2O
5	Valeric	CH_3-$(CH_2)_3$-COOH	5	102	Yes	-	CO_2, C_3H_6, CH_3COOH, HCOOH, CO
6	Hexanoic	CH_3-$(CH_2)_4$-COOH	6	116	Yes	C_2H_6	CO, H_2O
7	Decanoic	CH_3-$(CH_2)_8$-COOH	10	172	Yes	CO	H_2, CO, $(H_2+C_6H_{12})$, $(H_2+C_5H_{10})$, $(H_2 + H_2O)$, C_7H_{16}
8	Lauric	CH_3-$(CH_2)_{10}$-COOH	12	200	Yes (2.9 ppm)	-	H_2O, CO
9	Myristic	CH_3-$(CH_2)_{12}$-COOH	14	228	Yes	-	H_2O, CO
10	Palmitic	CH_3-$(CH_2)_{14}$-COOH	16	256	Yes	-	H_2O, $2H_2O$, CO
11	Stearic	CH_3-$(CH_2)_{16}$-COOH	18	284	Yes (2.0 ppm)	CO	H_2O, CO, $2H_2O$
12	Behenic	CH_3-$(CH_2)_{20}$-COOH	22	341	Yes (3.3 ppm)	-	H_2O, $2H_2O$, CO

Table S2. Full MS analysis and esterification of unsaturated fatty acids when using the vapors of methanol and carboxylic acids, in positive (+) and negative (-) plasma. Fragments are shown in order of decreasing MS relative intensity.

#	Acid	Formula	Carbon #	MW	Esterification	Fragments (+)
1	4-pentenoic	CH_2=CH-$(CH_2)_2$-COOH	5	100	No	HCOOH, CO, H_2O
2	4-hexenoic	CH_3-CH=CH-$(CH_2)_2$-COOH	6	114	No	H_2O
3	*trans*-3-hexenoic	CH_3-CH_2-CH=CH-CH_2-COOH	6	114	Yes	HCOOH, C_3H_6, CH_3COOH, H_2O
4	*trans*-2-hexenoic	CH_3-$(CH_2)_2$-CH=CH-COOH	6	114	Yes	CH_3COOH, C_3H_6, H_2O, HCOOH
5	5-Decenoic	CH_3-$(CH_2)_3$-CH=CH-$(CH_2)_3$-COOH	10	170	No	H_2O, $2H_2O$
6	6-Decenoic	CH_3-$(CH_2)_2$-CH=CH-$(CH_2)_4$-COOH	10	170	No	H_2O, $2H_2O$
7	*cis*-5-Dodecenoic	CH_3-$(CH_2)_5$-CH=CH-$(CH_2)_3$-COOH	12	198	No	H_2O, $2H_2O$
8	Palmitoleic	CH_3-$(CH_2)_5$-CH=CH-$(CH_2)_7$-COOH	16	254	No	H_2O, $2H_2O$
9	Linoleic	CH_3-$(CH_2)_4$-CH=CH-CH_2-CH=CH-$(CH_2)_7$-COOH	18	280	No	H_2O, $2H_2O$
10	Oleic	CH_3-$(CH_2)_7$-CH=CH-$(CH_2)_7$-COOH	18	282	No	H_2O, $2H_2O$
11	Vaccenic	CH_3-$(CH_2)_5$-CH=CH-$(CH_2)_9$-COOH	18	282	No	H_2O, $2H_2O$
12	Erucic	CH_3-$(CH_2)_7$-CH=CH-$(CH_2)_{11}$-COOH	22	339	No	H_2O, $2H_2O$

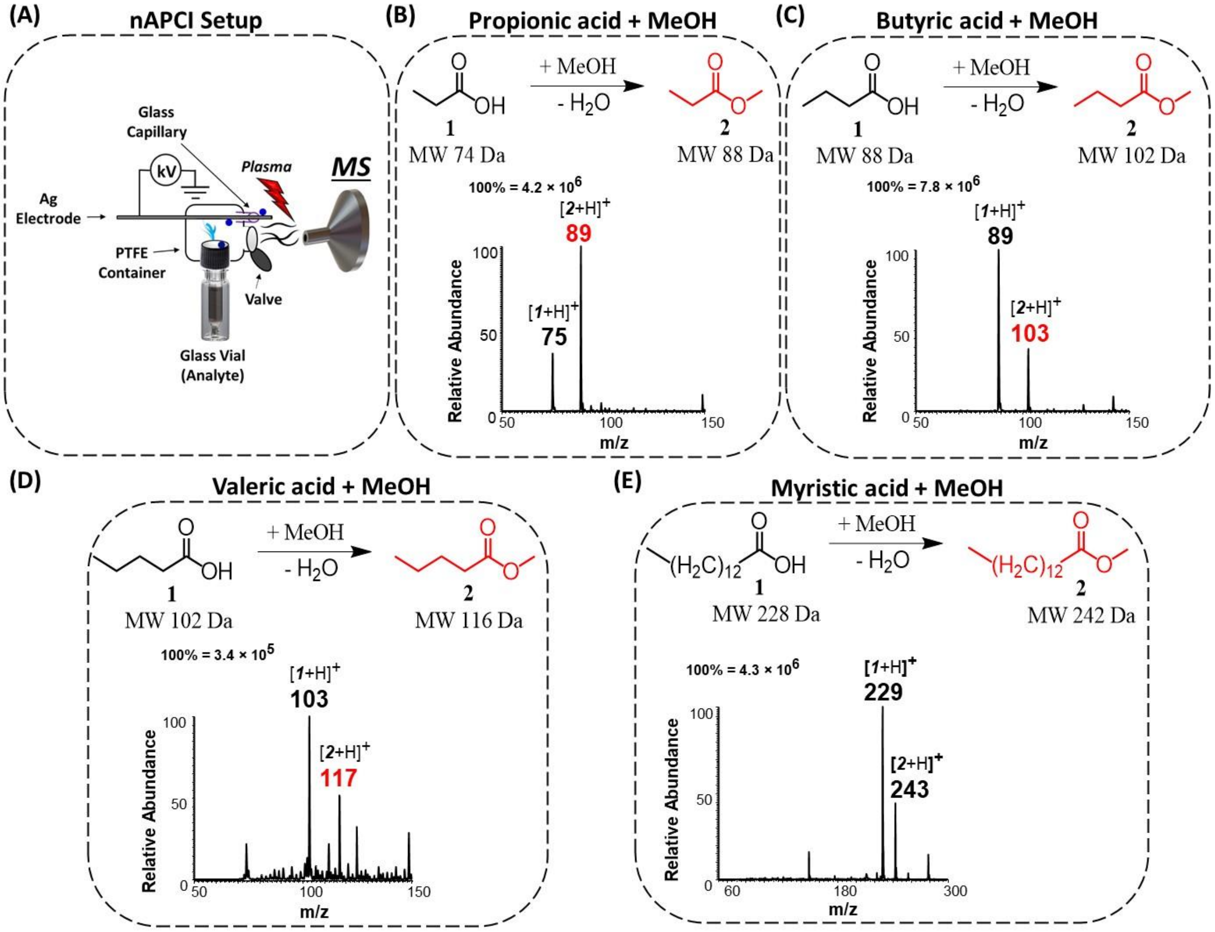

Fig. S1. (**A**) Setup for contained nano-atmospheric pressure chemical ionization mass spectrometry (nAPCI MS) used for analyzing headspace vapors of compounds.[1] Corona discharge is produced at the tip of the Ag electrode during application of DC voltage >4 kV. Potential also causes reactive olfaction to electrostatically attract/adsorb the solid particles of an analyte (in the glass vial) on the electrode surface with subsequent APCI ionization. The PTFE container concentrates the vapor-phase molecules/particles into a small space and the glass capillary collimates the vapor toward the plasma. The valve on the side of the PTFE container increases vapor flow rate by reducing the saturation of air above the sample. For esterification, the methanol vapor was brought in between the nAPCI source and the MS inlet. Positive-ion mode mass spectra recorded for esterification reactions between headspace vapors of methanol and saturated fatty acids: (**B**) propionic acid, (**C**) butyric acid, (**D**) valeric acid, and (**E**) myristic acid. Nano-ESI was not used here. The experimental setup for analyzing vapors is shown below.

3. Esterification of saturated fatty acids by ethanol

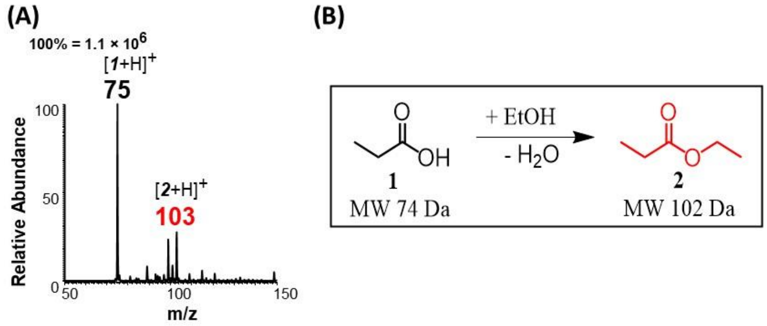

Fig. S2. Positive-ion mode mass spectrum derived from the analysis of acetic acid in the presence of ethanol headspace vapor, which reacted *via* esterification mechanism to give expected product at *m*/*z* 103. (**A**) Full MS spectrum recorded in real-time during a plasma desorption/ionization experiment and (**B**) schematic illustration of expected reaction.

4. Determination of the structure of [M+H+14]+ product

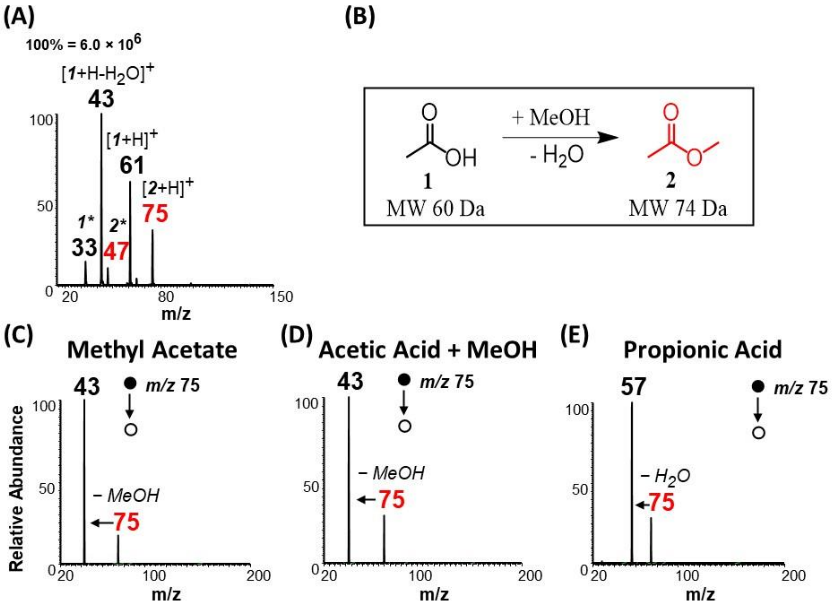

Fig. S3. Structure determination of [M+H+14]+ product headspace analysis of acid in the presence of methanol vapor. Two mechanisms were investigated: methylene insertion and esterification, both of which will add 14 Da to the reactant. For acetic acid, the expected products methyl acetate (*via* esterification) and propionic acid (*via* methylene insertion). We used standard of methyl acetate and propionic acid to compare with product formed in our experiment. (**A**) Full mass spectrum of gas-phase reaction recorded in real-time involving the use of vapors of acetic acid and methanol, (**B**) reaction scheme showing the formation of corresponding ester from acetic acid after exposure to methanol vapor. Tandem MS of (**C**) standard methyl acetate, (**D**) peak at *m*/*z* 75 in spectrum (**A**), which was generated from acetic acid / methanol reaction and (**E**) standard propionic acid. MS/MS spectrum from methyl acetate more closely resembles MS/MS derived from our online reaction, which excludes methylene insertion.

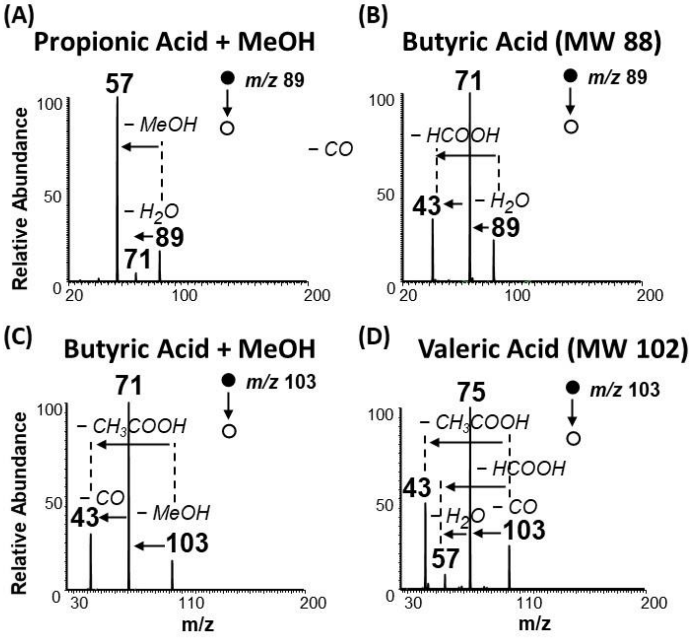

Fig. S4. Positive-ion mode MS/MS spectra of (**A**) reaction product (MW 88 Da) generated from plasma ionization of propionic acid in the presence of methanol vapor, (**B**) standard butyric acid, (**C**) reaction product (MW 102 Da) generated from analysis of butyric acid in the presence of methanol, and (**D**) standard of valeric acid. The data excludes methylene insertion, which will afford the carboxylic acid with one carbon longer than the reactant. The fragmentation pattern of the observed ester products (**A** and **C**) is markedly different from the corresponding acids of the same molecular weight (**B** and **D**).

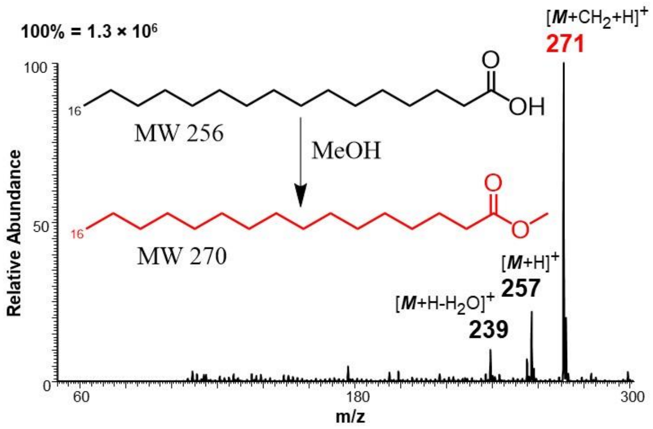

Fig. S5. Positive-ion mode mass spectrum of palmitic acid recorded in the presence of MeOH headspace vapor, after exposure to corona discharge.

5. Lactone formation from hydroxycarboxylic acids

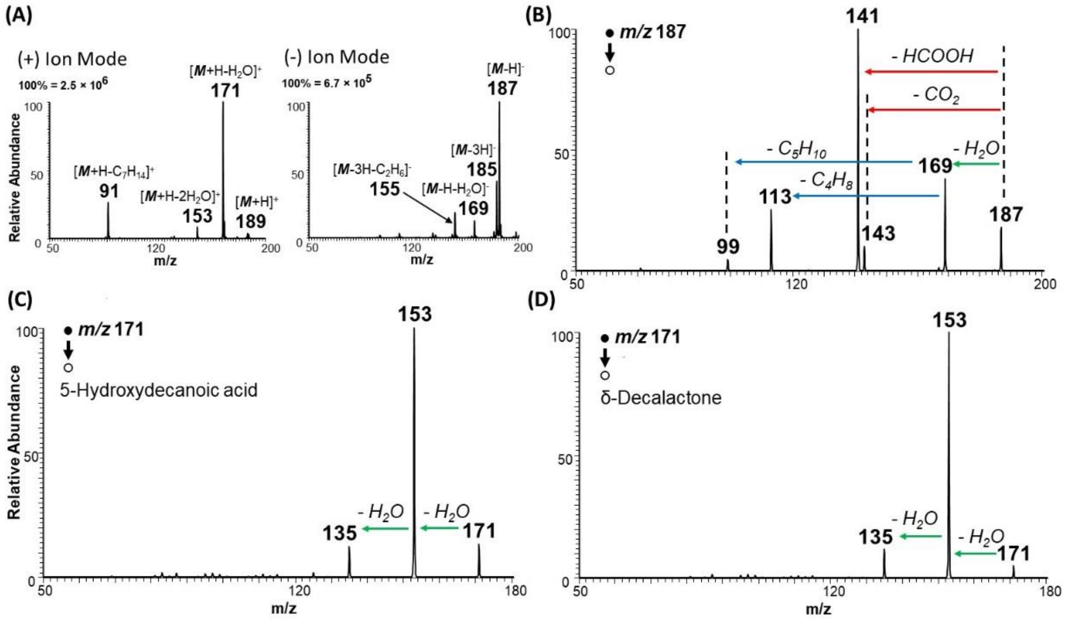

Fig. S6. Formation of δ-decalactone from 5-hydroxydecanoic acid standard under discharge conditions (no solvent) in (**A**) full MS positive ion mode (first spectrum) and negative ion mode (second spectrum). (**B**) MS/MS of deprotonated 5-hydroxydecanoic acid derived from (A) in negative-ion mode, where lactone formation was not observed. (**C**) MS/MS of $[M + H - H_2O]^+$ ions derived from 5-hydroxydecanoic acid standard generated from (A) in positive-ion mode and (**D**) MS/MS of δ-decalactone standard.

The geometry optimization of the reagent, products and all intermediates along the transformation pathway was caried out by using B3LYP functional,[2,3] 6-311g(d,p)[4,5] basis and empirical dispersion at GD3[6] level. All calculations were performed in a gas phase approximation. The energetics along transformation mechanism was calculated with accounting of zero point energy (ZPE) and Gibbs free energy corrections. All the simulations were realized by using Gaussian16 software.[7]

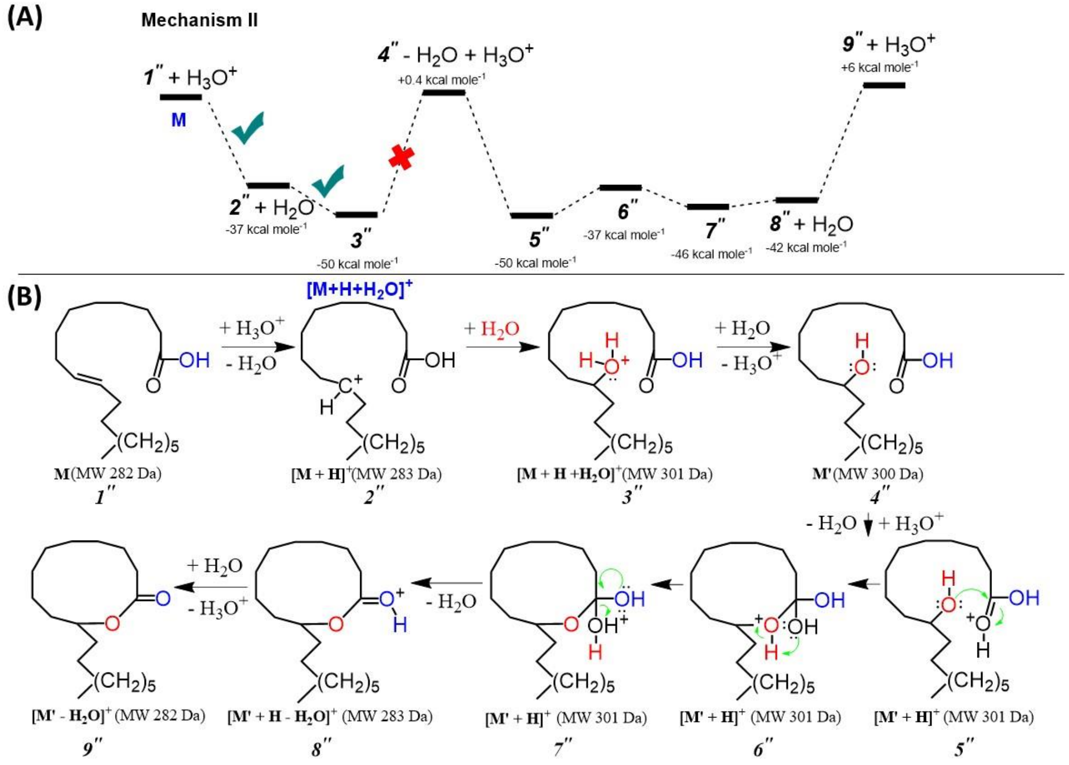

Fig. S7. (**A**) DFT free energy diagram of (**B**) gas-phase lactone (***9’’***) synthesis from oleic acid (***1’’***) through intermediate hydroxycarboxylic acid (***4’’***) formation.

6. Proton affinity discussion and calculations

Differences in proton affinity ruled out. Since protonation is the crucial step in Fisher esterification, our next investigation was related to probable difference in proton affinities for unsaturated *versus* saturated FAs. Unfortunately, the literature does not provide sufficient information about proton affinities of FAs to make any informed conclusions. However, it is known that measured pKa values in solution increase with increasing FA chain length but decrease with increasing number of double bonds.[8] If we assume the same acidity/basicity trend for gas phase species, then proton affinities of unsaturated FAs should be less than saturated species, which may prevent their protonation and subsequent esterification. Intuitively, we can expect a higher C=C bond influence on carboxyl group acidity when it is closest. But as we discussed above, proximity of double bond has the opposite effect on esterification; it is favored. Moreover, hydronium ions (PA 725.6 kJ/mol), thought to be present in our experiment, have sufficient ability to protonate any fatty acid, including the strongest carboxylic acids such as formic acid (PA 742.0 kJ/mol), acetic acid (PA 783.7 kJ/mol), and propanoic acid (PA 797.2 kJ/mol). Thus, it is unreasonable to believe that differences in proton affinities, if any, can suppress esterification of unsaturated FAs.

Proton affinities (PA) were calculated according to formula:

$$\textbf{PA} = -(\Delta H_f(\text{Protonated Compound}) - [\Delta H_f(\text{Compound}) + \Delta H_f(H^+)])$$

where ΔH_f is standard enthalpy of formation for the corresponding species.

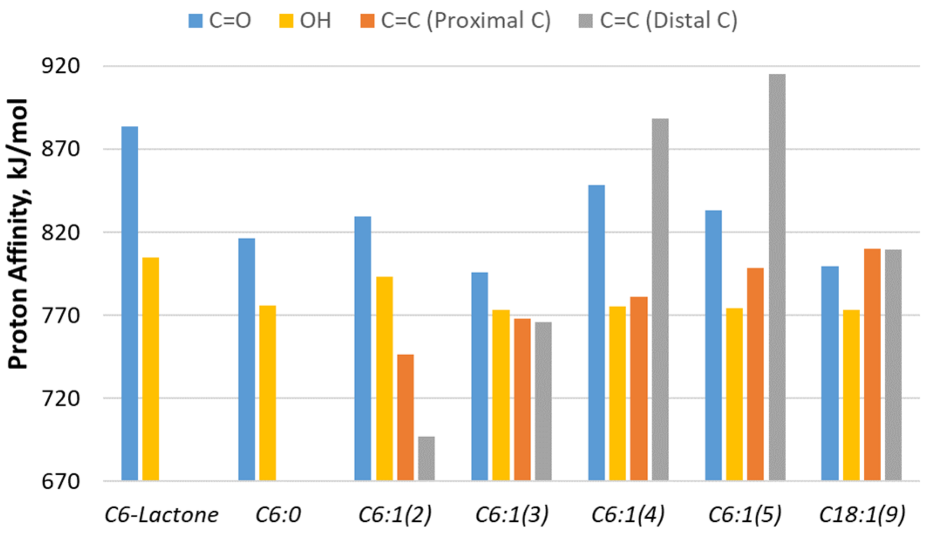

Fig. S8. DFT calculations (B3LYP/6-311+G(2d,p)) for proton affinities of lactone group, carbonyl group, carbon-carbon double bond (at proximal and distal carbon relatively to carboxyl group) and hydroxyl group of hexanoic acid (C6:0), hexenoic acids with different positions of double bond (after C_2, C_3, C_4, and C_5), and oleic acid. Computational details are described in **Section 5**.

7. Fisher esterification mechanism

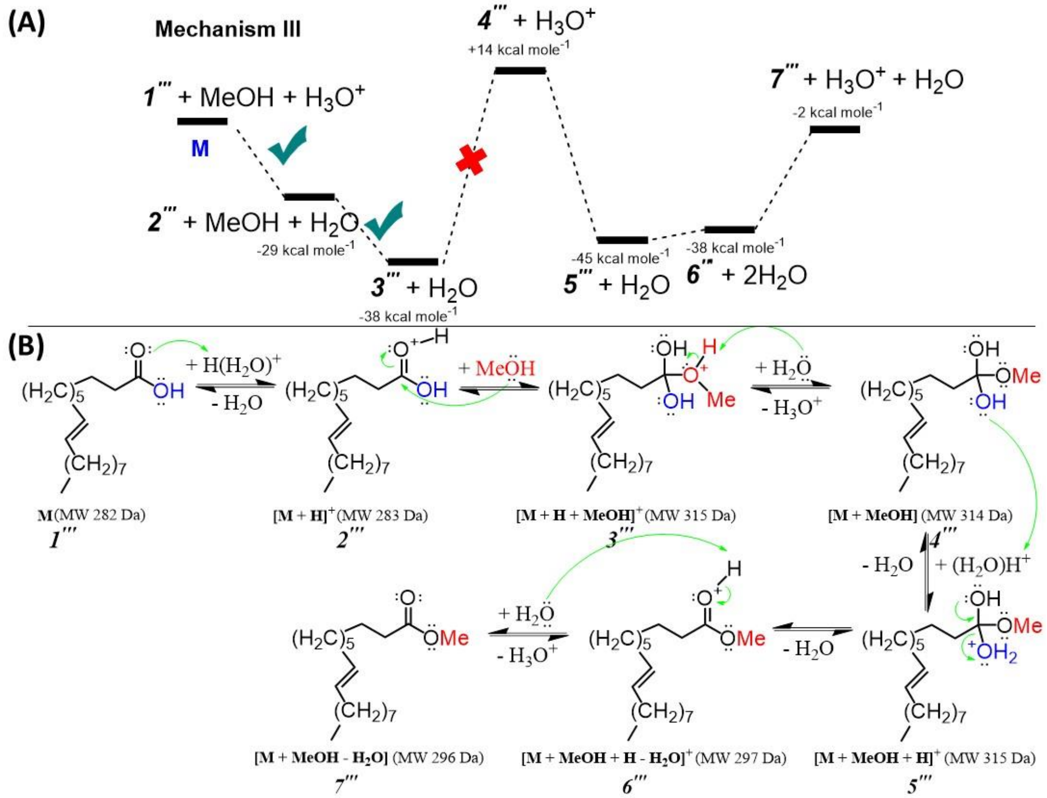

Fig. S9. (**A**) DFT free energy diagram of (**B**) gas-phase Fisher esterification of oleic acid (***1''***) by methanol. Computational details are described in **Section 5**.

8. Intramolecular cyclization of unsaturated fatty acids

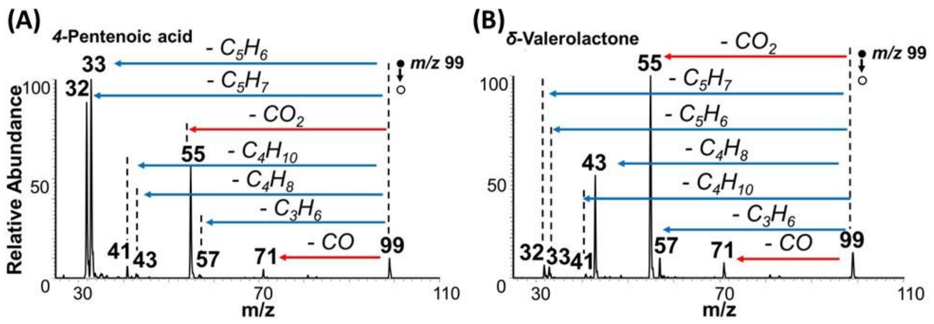

Fig. S10. Tandem MS data in negative-ion mode for (**A**) 4-pentenoic acid and (**B**) δ-valerolactone obtained analyzed using headspace vapors of the acids in the presence of nonthermal plasma.

9. Plasma fragmentation of saturated and unsaturated fatty acids

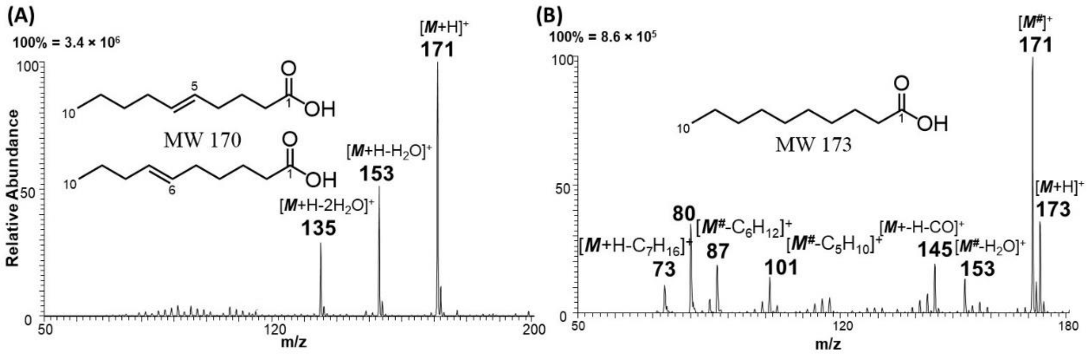

Fig. S11. Full MS fragmentation of (**A**) mixture of 5-decenoic and 6-decenoic acids, and (**B**) decanoic acid analyzed in the presence of nonthermal plasma. Unsaturated acids consistently loose water molecules.

10. Isobaric overlap experiments

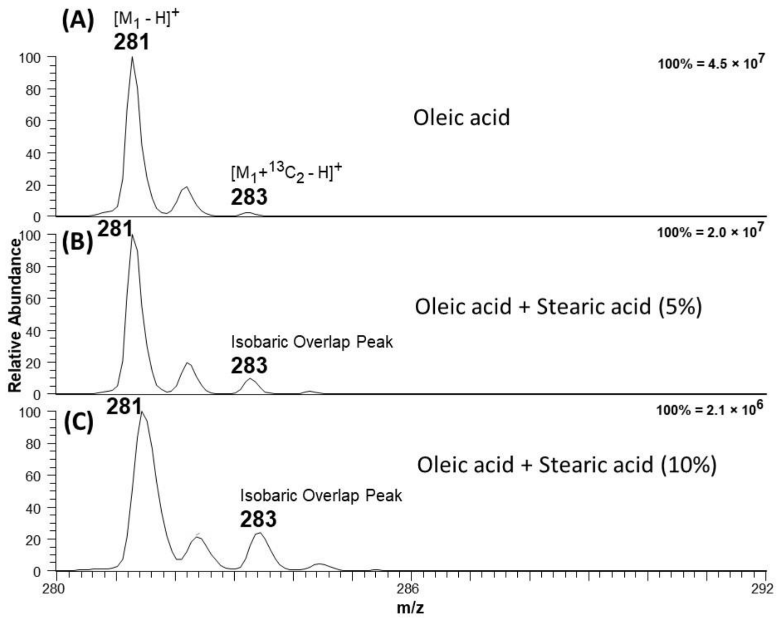

Fig. S12. Negative-ion mode mass spectra showing analysis of methanolic solutions of (**A**) 500 µM oleic acid, (**B**) 500 µM oleic and 25 µM stearic acid, and (**C**) 500 µM oleic and 50 µM stearic acid, all achieved *via* plasma-nanodroplet fusion.

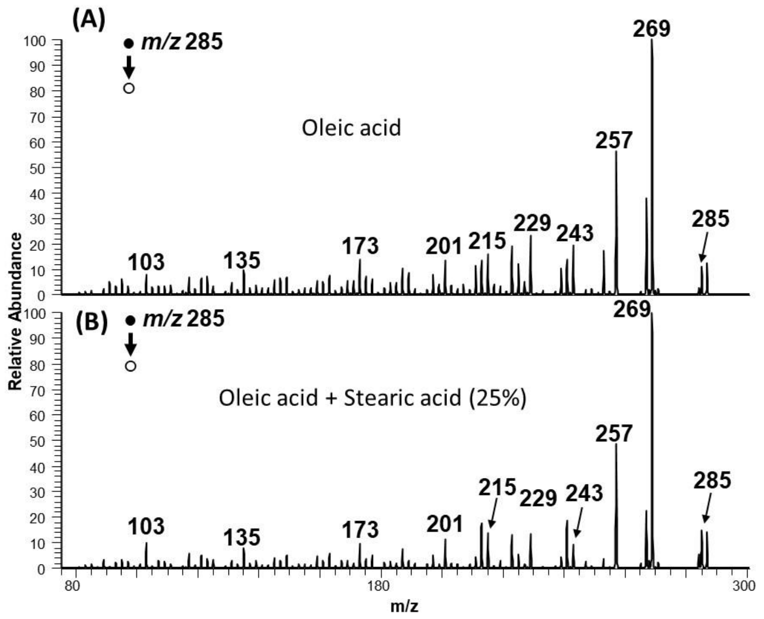

Fig. S13. Positive-ion mode MS/MS spectra of (**A**) oleic acid (500 µM) and (**B**) oleic (500 µM)/stearic (125 µM) acids mixture at *m/z* 285 position where isobaric overlap occurs. It could be difficult to infer the presence of stearic acid without shifting the mass to another *m/z* position.

11. Calibration curve for online esterification

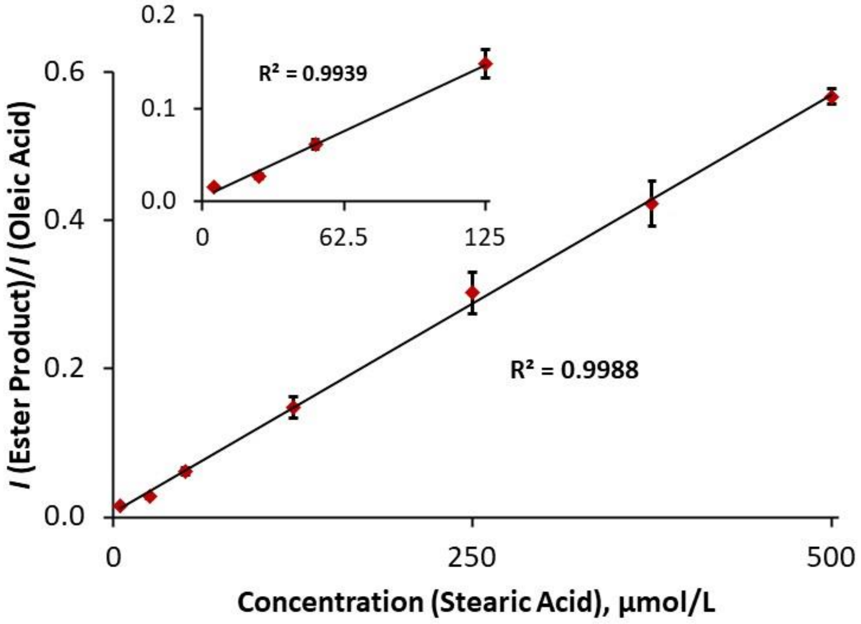

Fig. S14. Calibration curve for online esterification of stearic acid in presence of oleic acid in methanolic solvent where the methyl stearate ion (*m/z* 299) intensity at different concentrations of stearic acid analyte (5 – 500 µmol/L) was compared to that of oleic acid (500 µmol/L, *m/z* 283).

12. CID fragmentation of fatty acids

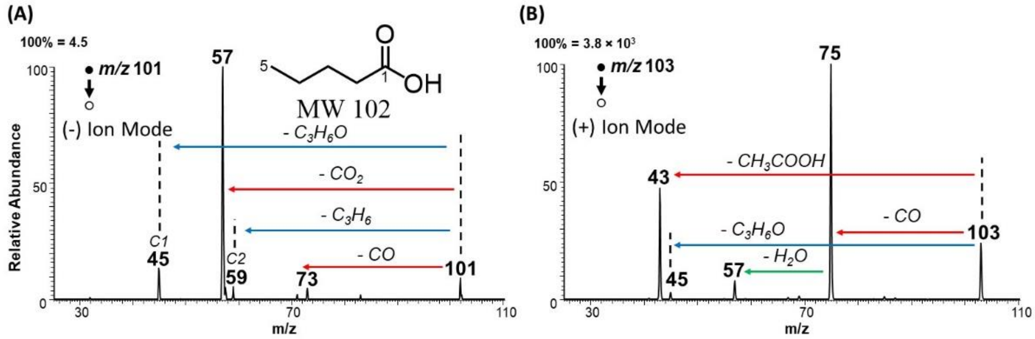

Fig. S15. Tandem MS data for valeric acid (**A**) in negative and (**B**) positive ion modes.

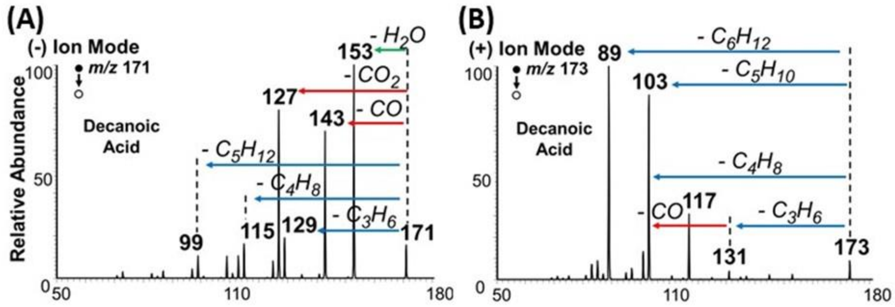

Fig. S16. MS/MS spectra of decanoic acid (**A**) in negative and (**B**) positive ion modes.

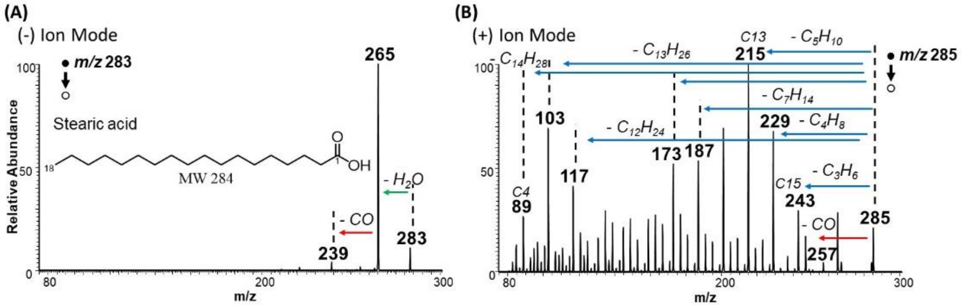

Fig. S17. MS/MS data for stearic acid (**A**) in negative and (**B**) positive ion modes.

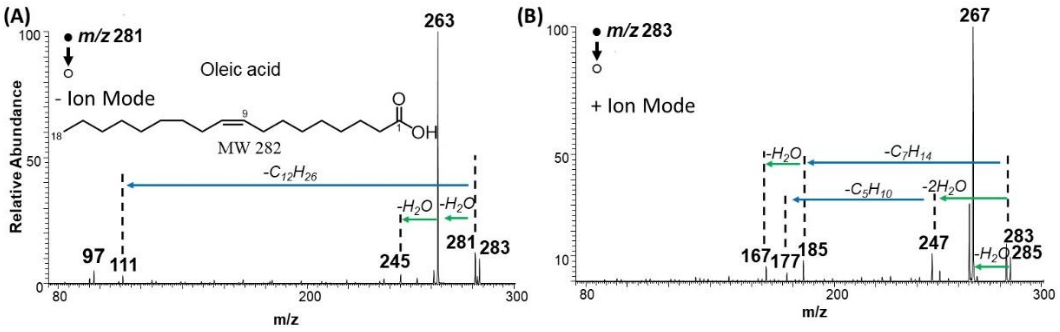

Fig. S18. Tandem MS data for oleic acid (**A**) in negative and (**B**) positive ion modes.

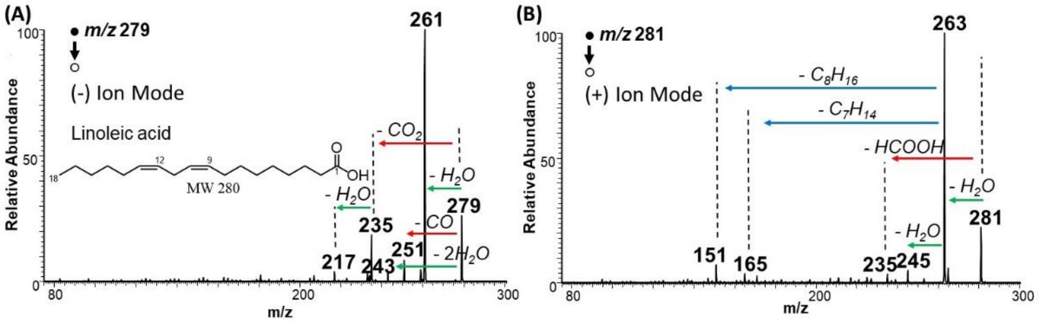

Fig. S19. MS/MS spectra of linoleic acid (**A**) in negative and (**B**) positive ion modes.

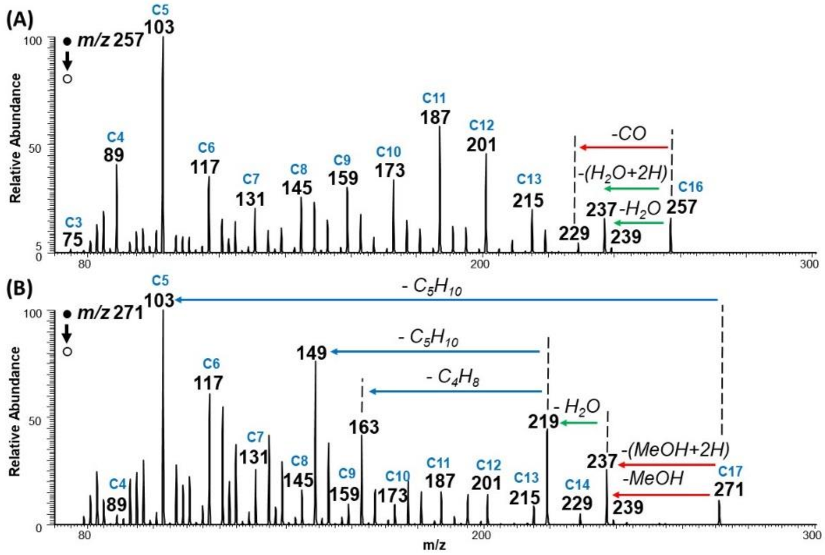

Fig. S20. Positive-ion mode MS/MS data for (**A**) palmitic acid and (**B**) its methyl ester.

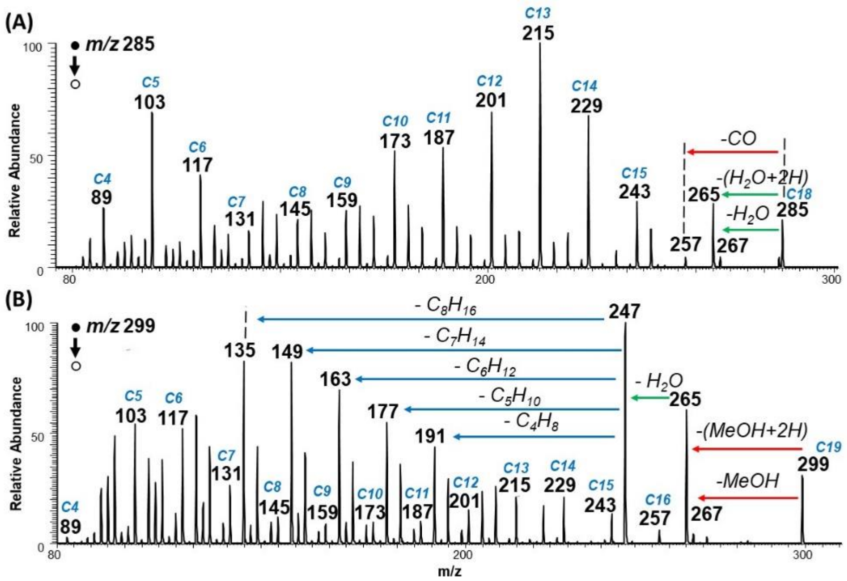

Fig. S21. Positive-ion mode tandem MS data for (**A**) stearic acid and (**B**) its methyl ester.

13. Coconut oil analysis

Coconut oil and hydrolyzed coconut oil were diluted in methanol (1:1000) prior to analysis. The hydrolysis of coconut oil was performed by solution of KOH in ethanol. 2.5 mL of the melted oil was mixed with 3.0 mL of 15% KOH in ethanol and then heated till all ethanol evaporated. The hydrolyzed oil was then diluted in hot ethanol and finally in methanol to give 1:1000 final concentration.

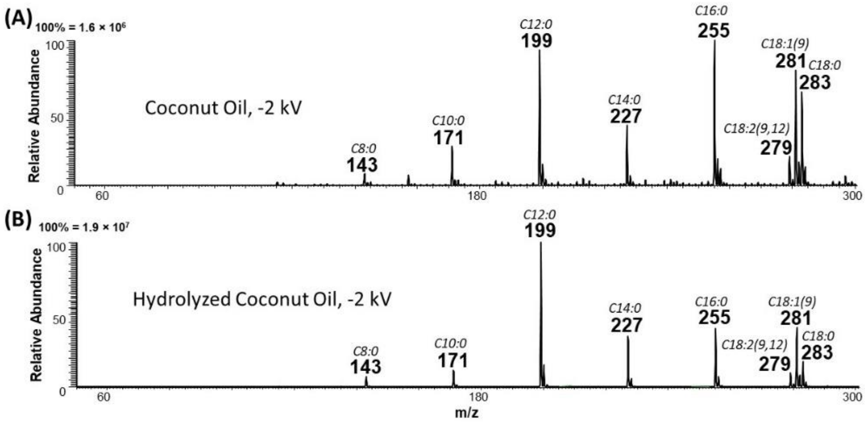

Fig. S22. Negative-ion mode mass spectra of (**A**) diluted coconut oil and (**B**) hydrolyzed coconut oil at **-** 2 kV nano-ESI, in the absence of plasma.

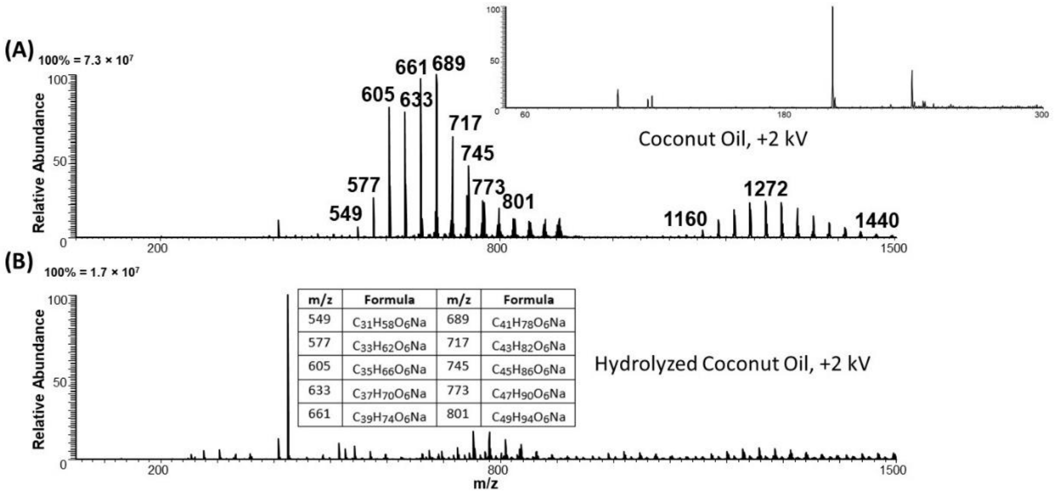

Fig. S23. Positive-ion mode mass spectra of (**A**) diluted coconut oil and (**B**) hydrolyzed coconut oil at 2 kV nano-ESI, in the absence of plasma. Insert in **(A)** shows absence of fatty acids at lower *m/z* range in positive-ion mode at low spray voltage. Insert in (B) shows molecular formula assignment based on previous report,[9] which also detected the same peaks.

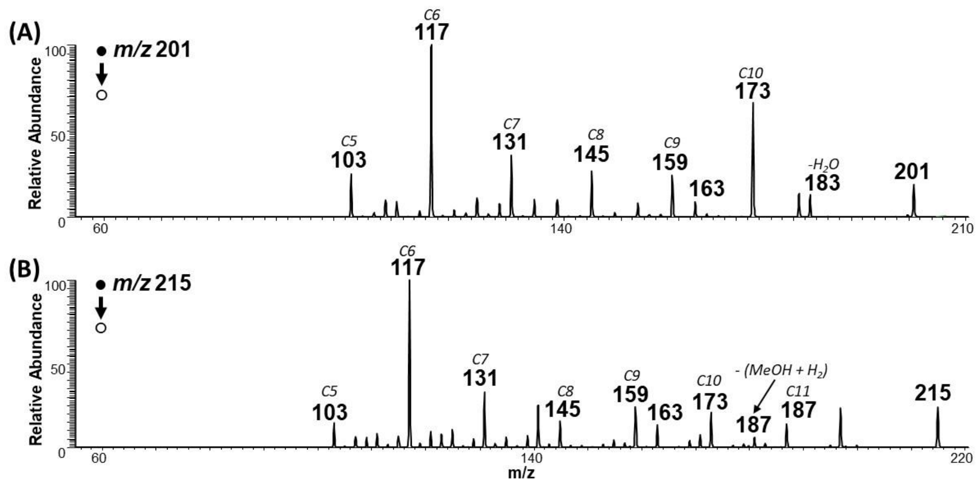

Fig. S24. Positive-ion mode tandem MS data for (**A**) lauric acid and (**B**) methyl laurate, originating from coconut oil, when analyzed with plasma-nanodroplet fusion at +6 kV.

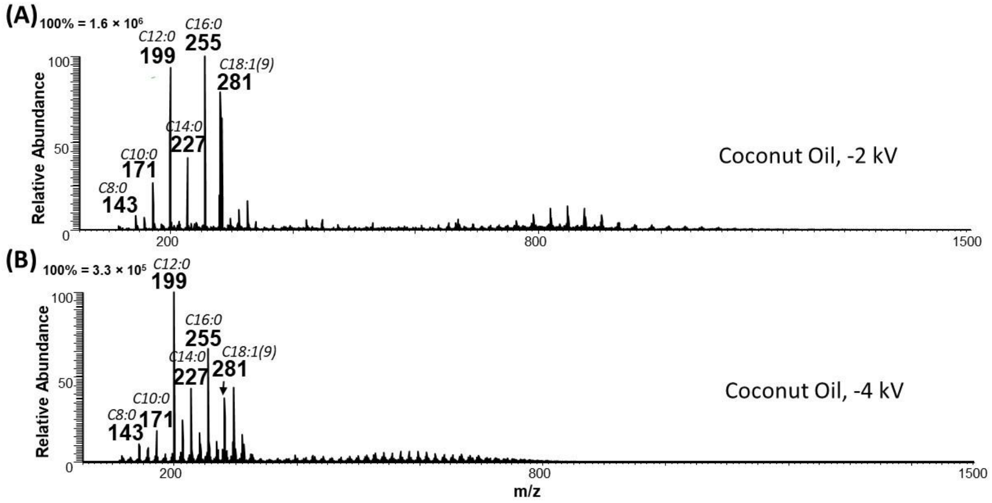

Fig. S25. Negative-ion mode MS analysis of Coconut oil using (**A**) – 2 kV, in the absence of plasma and (**B**) – 4 kV in the presence of plasma. Negative plasma fusion into negative droplets do not result in any noticeable change in mass spectra.

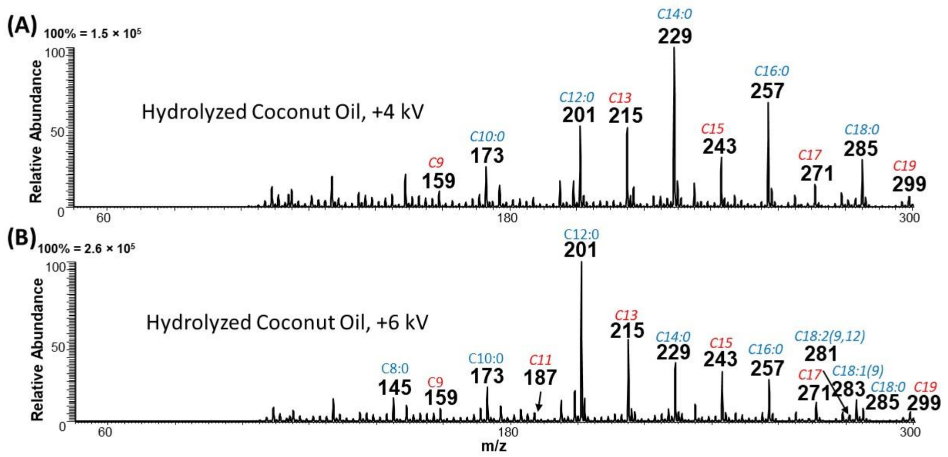

Fig. S26. Positive-ion mode MS analysis of hydrolyzed coconut oil at (**A**) 4 kV and (**B**) 6 kV, all in the presence of plasma with +6 kV showing more reactive droplets for esterification. Peaks labeled with red font are ester products of the corresponding acid [C9 from C8:0 and C13 from C12:0]. Literature confirms the odd number saturated fatty acids are not found in coconut oil.[10]

14. References

[1] D. S. Kulyk, T. Sahraeian, Q. Wan, A. K. Badu-Tawiah, *Anal. Chem.* **2019**, *91*, 6790–6799.

[2] A. D. Becke, *The Journal of Chemical Physics* **1993**, *98*, 5648–5652.

[3] C. Lee, W. Yang, R. G. Parr, *Phys. Rev. B* **1988**, *37*, 785–789.

[4] A. D. McLean, G. S. Chandler, *The Journal of Chemical Physics* **2008**, *72*, 5639–5648.

[5] M. J. Frisch, J. A. Pople, J. S. Binkley, *The Journal of Chemical Physics* **1984**, *80*, 3265–3269.

[6] S. Grimme, J. Antony, S. Ehrlich, H. Krieg, *J Chem Phys* **2010**, *132*, 154104.

[7] Gaussian 16, Revision B.01, M. J. Frisch, G. W. Trucks, H. B. Schlegel, G. E. Scuseria, M. A. Robb, J. R. Cheeseman, G. Scalmani, V. Barone, G. A. Petersson, H. Nakatsuji, X. Li, M. Caricato, A. V. Marenich, J. Bloino, B. G. Janesko, R. Gomperts, B. Mennucci, H. P. Hratchian, J. V. Ortiz, A. F. Izmaylov, J. L. Sonnenberg, D. Williams-Young, F. Ding, F. Lipparini, F. Egidi, J. Goings, B. Peng, A. Petrone, T. Henderson, D. Ranasinghe, V. G. Zakrzewski, J. Gao, N. Rega, G. Zheng, W. Liang, M. Hada, M. Ehara, K. Toyota, R. Fukuda, J. Hasegawa, M. Ishida, T. Nakajima, Y. Honda, O. Kitao, H. Nakai, T. Vreven, K. Throssell, J. A. Montgomery, Jr., J. E. Peralta, F. Ogliaro, M. J. Bearpark, J. J. Heyd, E. N. Brothers, K. N. Kudin, V. N. Staroverov, T. A. Keith, R. Kobayashi, J. Normand, K. Raghavachari, A. P. Rendell, J. C. Burant, S. S. Iyengar, J. Tomasi, M. Cossi, J. M. Millam, M. Klene, C. Adamo, R. Cammi, J. W. Ochterski, R. L. Martin, K. Morokuma, O. Farkas, J. B. Foresman, and D. J. Fox, Gaussian, Inc., Wallingford CT, 2016.

[8] A. A. Pashkovskaya, M. Vazdar, L. Zimmermann, O. Jovanovic, P. Pohl, E. E. Pohl, *Biophys J* **2018**, *114*, 2142–2151.

[9] J. A. Ferreira, J. M. Santos, M. C. Breitkreitz, J. M. S. Ferreira, P. M. P. Lins, S. C. Farias, D. R. de Morais, M. N. Eberlin, C. B. G. Bottoli, *Food Res Int* **2019**, *123*, 189–197.

[10] W. M. de Azevedo, L. F. R. de Oliveira, M. A. Alcântara, A. M. T. de M. Cordeiro, K. S. F. da S. C. Damasceno, N. K. de Araújo, C. F. de Assis, F. C. de S. Junior, *PLOS ONE* **2020**, *15*, e0232224.
